# Reprogramming of phytopathogen transcriptome by a non-bactericidal pesticide residue alleviates its virulence in rice

**DOI:** 10.1016/j.fmre.2021.12.012

**Published:** 2022-01-20

**Authors:** Haruna Matsumoto, Yuan Qian, Xiaoyan Fan, Sunlu Chen, Yanxia Nie, Kun Qiao, Dandan Xiang, Xinzhong Zhang, Meng Li, Bo Guo, Peilin Shen, Qiangwei Wang, Yunlong Yu, Tomislav Cernava, Mengcen Wang

**Affiliations:** a State Key Laboratory of Rice Biology & Ministry of Agricultural and Rural Affairs Laboratory of Molecular Biology of Crop Pathogens and Insects, Institute of Pesticide and Environmental Toxicology, Zhejiang University, Hangzhou 310058, China; b Key Laboratory of Biology of Crop Pathogens and Insects of Zhejiang Province, College of Agriculture and Biotechnology, Zhejiang University, Hangzhou 310058, China; c Institute of Environmental Biotechnology, Graz University of Technology, Petersgasse 12, 8010 Graz, Austria; d Global Education Program for AgriScience Frontiers, Graduate School of Agriculture, Hokkaido University, Sapporo 060-8589, Japan; e State Key Laboratory of Crop Genetics and Germplasm Enhancement, Nanjing Agricultural University, Nanjing 210095, China; f Ecology and Environmental Sciences Center, South China Botanical Garden, Chinese Academy of Sciences, Guangzhou 510650, China; g Key laboratory of South Subtropical Fruit Biology and Genetic Resource Utilization (MOA), Institute of Fruit Tree Research, Guangdong Academy of Agricultural Sciences, Guangzhou 510640, China; h Tea Research Institute, Chinese Academy of Agricultural Sciences, Hangzhou 310008, China; i Shanghai International Studies University, Shanghai 200083, China; j Xiaoshan Agricultural Comprehensive Development Zone & Management Committee, Hangzhou 311200, China

**Keywords:** Phytopathogen, Virulence factor, Transcriptome reprogramming, Agrochemical, Pesticide, Rice

## Abstract

Bacteria equipped with virulence systems based on highly bioactive small molecules can circumvent their host's defense mechanisms. Pathogens employing this strategy are currently threatening global rice production. In the present study, variations in the virulence of the highly destructive *Burkholderia plantarii* were observed in different rice-producing regions. The environment-linked variation was not attributable to any known host-related or external factors. Co-occurrence analyses indicated a connection between reduced virulence and 5-Amino-1,3,4-thiadiazole-2-thiol (ATT), a non-bactericidal organic compound. ATT, which accumulates in rice plants during metabolization of specific agrochemicals, was found to reduce virulence factor secretion by *B. plantarii* up to 88.8% and inhibit pathogen virulence by hijacking an upstream signaling cascade. Detailed assessment of the newly discovered virulence inhibitor resulted in mechanistic insights into positive effects of ATT accumulation in plant tissues. Mechanisms of virulence alleviation were deciphered by integrating high-throughput data, gene knockout mutants, and molecular interaction assays. TroK, a histidine protein kinase in a two-component system that regulates virulence factor secretion, is likely the molecular target antagonized by ATT. Our findings provide novel insights into virulence modulation in an important plant-pathogen system that relies on the host's metabolic activity and subsequent signaling interference.

## Graphical abstract

**Figure:**
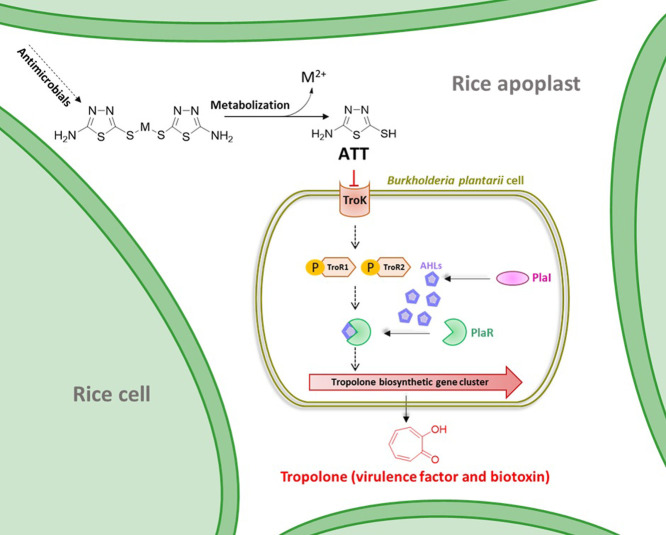

## 1 Introduction

Rice paddies, which constitute the largest artificial agro-ecosystem on Earth, provide one of the main food resources for a growing global population [1,2]. Being grown in warm and humid regions, rice plants often suffer from several biotic stresses [2], [3], [4] and are affected by prevalence of destructive bacterial pathogens that have emerged as an increasingly severe threat for global rice production [5]. Since the past three decades, *Burkholderia* pathogens with highly efficient virulence factors have spread across Asia [6], [7], [8], [9], Africa [10] and America [11,12]. As a typical destructive species, *Burkholderia plantarii* infects host plants covering rice and another twelve genera of gramineae crops [6,13,14].

Virulence factor expression plays a pivotal role in successful infection and multiplication of bacterial pathogens inside their host plants [15,16]. During host infection, *B. plantarii* secretes tropolone as a major virulence factor to cause seedling or panicle blight [6]. Due to a broad spectrum of cytotoxicity to mammals and aquatic organisms, tropolone is also an important biotoxin that must be considered in terms of environmental risks and food safety [17,18]. Local accumulation of tropolone is known to favor survival and dispersal of *B. plantarii* through biofilm development in response to hostile environments and to overcome host innate immunity.

The innate and acquired immune systems of plants have primarily evolved to recognize macromolecule-type virulence factors (e.g. cellular surface-carbohydrates and proteins) [19,20]. Immune responses are often connected to PAMP-triggered immunity (PTI) and effector-triggered immunity (ETI) in coordination with complex signaling cross-talk [21]. However, commonly employed defense strategies remain ineffective when challenged by pathogens armed with small-molecule-type virulence factors, such as tropolone. Recently, accumulating evidence obtained in the context of the phytobiome suggests that the environment has an underestimated role in plant-microbe interactions [22]. Further insights into the underlying effects of pathogen inhibition at molecular level might provide a basis for upcoming solutions for plant protection. Such solutions could make use of on *in situ* regulation of small-molecule-based virulence factors secreted by highly destructive bacterial pathogens [23], [24], [25].

Currently, agricultural practices in rice cultivation mainly rely on high inputs of agrochemicals to prevent the spread of pathogens at a global scale [26]. Most of the applied antimicrobials are mainly used for the prevention of fungal diseases and rarely target bacterial phytopathogens. In China, public registries indicate region-specific uses of various antimicrobials, which include the chemical classes of triazole, strobilurin, aminoglycosides, benzimidazoles, thiazoles, amides, and organic sulfurs [1,[27], [28], [29], [30]]; however, none of them is specifically applied against *Burkholderia* pathogens. Unexpectedly, a pilot study covering all major rice growing areas in China found that virulence of *B. plantarii* varied widely in geographically separated cultivation sites in Zhejiang Province [17]. It was previously described that agrochemicals can be absorbed by various plant tissues and metabolized to trace levels, along with formation of stable metabolites in distinct intercellular microenvironments [2,31]. So far, the impact of plant-transformed agrochemicals on highly dynamic pathogen-host interactions and more specifically on *in situ* virulence factor expression by phytopathogens remained largely unexplored.

In order to investigate potential implications, we systematically profiled antimicrobials including parent molecules as well as plant-metabolized products in geographically separated rice cultivation regions with simultaneous detection of the pathogen's virulence factor occurrence in rice spikelets. Due to a remarkable negative co-occurrence, we focused on the effects of thiazole-class antimicrobials on the virulence of pathogens in rice plants. By integration of high-throughput data, gene knockout mutants, biochemical analyses, molecular interaction data and field trials, mechanistic insights into the virulence-alleviating effect of a non-bactericidal metabolization product that was derived from a thiazoles-class antimicrobial and accumulated in plant tissues were unveiled. This work highlights a so far unknown biological role of a virulence modulator in rice cultivation to counteract bacterial pathogen infection.

## 2 Materials and methods

### 2.1 Chemicals, reagents and instruments

The main chemicals, reagents and instruments used in this study are listed here. Ethylicin, kasugamycin and validamycin (National Research Center for Certified Reference Materials, China); zinc thiazole (2-Amino-5-mercapto-1,3,4-thiadiazole zinc) and 20% zinc thiazole suspension concentrate (Zhejiang XinNong Chemical Co., China); other utilized standards for antimicrobial compounds (J&K, China); zinc sulfate (Sinopharm, China); 5-Amino-1,3,4-thiadiazole-2-thiol (ATT; Macklin, China); tropolone (TCI, Japan); Antibiotics, isopropyl β-D-1-thiogalactopyranoside (IPTG), acetonitrile, dichloromethane, ethyl acetate, methanol and other organic solvents (chromatographic grade; Merck, China); DisQuE Kit (Waters, USA); ABI Prism 310 Genetic Analyzer (Applied Biosystems, CA, USA); StepOnePlus Real-Time PCR thermal cycling block (Applied Biosystems); GC (Shimadzu GC-2010, Japan); HPLC (Shimadzu LC-20A, Japan), UPLC-MS/MS (Waters Quattro Premier XE, USA), GC-MS/MS (Agilent 7000C); Surface Plasmon Resonance (SPR, Biacore T200 system, GE Healthcare, USA).

### 2.2 Microbial strains, culture conditions and media

All strains were previously isolated and identified in our laboratory at Zhejiang University. *Burkholderia plantarii* ZJ171 and *Ralstonia solanacearum* ZJ30 were routinely grown in MA agar (NH_4_H_2_PO_4_, 1 g; KCl, 0.2 g; MgSO_4_•7H_2_O, 0.2 g; glucose, 10 g; ultrapure water, 1 L [pH adjusted to 6.2]) at 25 °C (37 °C for *Escherichia coli*) in the dark. The fungal pathogen *Rhizoctonia solani* ZJ29 was grown routinely in potato dextrose agar (PDA), to which 1.5% agar powder (Solarbio, Beijing) was added. Other media such as Luria Bertani (LB), Modified Winogradsky (MW), Mueller-Hinton (MH), Murashige & Skoog (MS) or their agar- or gellan gum-solidified media were also alternatively applied when necessary.

### 2.3 Field sampling and survey

Virulence of *B. plantarii* varied widely in representative rice fields in the six selected rice-growing areas of Zhejiang Province during a long-term assessment between 2012 and 2016 [17]. According to a complementary assessment of regionally applied antimicrobials (parent molecule residues or relevant metabolites) in rice plants grown in Zhejiang Province, seven classes of compounds that were widely applied were identified (Fig. S1) and thus hypothesized to be associated with varying tropolone secretion by *B. plantarii* in rice. Based on the aforementioned assessments, rice spikelets were collected from representative fields located in the six rice cultivation regions of Zhejiang Province in 2017 (Fig. S2). In each region, ten samples (whole spikelets; 1 kg per sample) were collected from adjacent fields using a random sampling approach. The samples were transported in an isolated box and dry ice to the laboratory within 6 h and then stored at −20 °C until extraction and mass-spectrometric (MS) quantification of tropolone and the antimicrobials. All field procedures were conducted in accordance to the guideline of Good Agricultural Practices issued by the Ministry of Agriculture of China and without the involvement of any endangered wildlife species or protected areas of land.

### 2.4 Profiling of antimicrobials and virulence factor expression in rice

To detect and quantify frequently applied antimicrobials in rice production, known agrochemicals were selected for the targeted approach (Fig. S1). The two polar aminoglycosides kasugamycin and validamycin were extracted and detected using a method based on UPLC-MS/MS as previously reported [27]. The QuEChERS approach [2] was applied for simultaneous extraction and the detection of carbendazim [32], ethylicin [28], trifloxystrobin [1], thifluzamide [3], tebuconazole [3], tricyclazole [33], triadimefon [33]; it was conducted according to previous reports. 5-Amino-1,3,4-thiadiazole-2-thiol (ATT) is a ubiquitous metabolization product of all thiazole-class antimicrobials in plants, therefore ATT was extracted as previously reported and quantified by UPLC-MS/MS to represent thiazole-class antimicrobials (analytical parameters shown in Tables S1&S2) [34]**.** The extraction and quantification procedure were validated by a recovery test and showed average recoveries ranging from 69-112% with relative standard deviations of 0.8-13.0% at different spike levels (Table S3). Virulence factor expression of *B. plantarii* in rice was analyzed by GC-MS/MS for quantification of tropolone after extraction using the DisQuE kit as previously reported [17].

### 2.5 Assessments of variation in antimicrobial effects towards typical rice pathogens

To investigate the effects of antimicrobials that potentially inhibit tropolone secretion, a 96-well plate bioassay was carried out to determine and compare their minimum inhibitory concentration (MIC) towards *B. plantarii*. The plant pathogen was cultured in MA overnight then collected by centrifugation and transferred into Mueller-Hinton (MH) media. It was cultured at 25 °C until the stationary phase was reached and adjusted to a final density of 10^8^ CFU/mL. After dilution with MH broth in a 1:1000 ratio, 100 μL of the diluted culture was transferred to 96-well plates with 100 μL antimicrobial standard solution in each well. The final concentrations were 288, 256, 192, 128, 64, 32, 16, 8, 4, 2, 1, 0.5, 0.25 and 0.125 μg**/**mL. For a complete evaluation of the MIC, the same procedure was also conducted with LB and MWB. Average mean values were calculated for the MIC of each antimicrobial tested. In addition, MICs of the antimicrobials against *R. solanacearum* and *R. solani* were also assessed with the same protocol. Phylogenetic comparison of trehalase among *B. plantarii, R. solanacearum* and *R. solani* was done by phylogenetic analysis using MEGA 7.0 [35]. For confirmation of trehalase gene occurrence, genomic DNA was extracted from each of the phytopathogens with MiniBEST Bacteria Genomic DNA Extraction Kit (TaKaRa, Japan) and Fungi Genomic DNA Extraction Kit (Solarbio, China), respectively, and amplified with specific primer pairs list in Table S4 before Sanger sequencing.

### 2.6 Effect of thiazole-class antimicrobials on rice pathosystem

To investigate the impact of thiazole-class antimicrobials, zinc thiazole was selected as the representative substance to test its effect in the rice pathosystem. Inoculation with *B. plantarii* was conducted at two vulnerable stages of rice (cv. Xiushui 09) cultivation, including the seedling stage and the flowering stage, during which zinc thiazole was applied. Briefly, surface-sterilized seeds were incubated for germination and subjected to treatment with zinc thiazole (0.05 mM) and *B. plantarii* (10^6^ CFU/mL), or *B. plantarii* (10^6^ CFU/mL), or zinc thiazole (0.05 mM), or sterile water. The treated rice seeds were then transplanted into a 10-cm (height) glass dish (10 seeds per dish) containing 25 ml of MS media w/vitamins (1/2 strength) solution solidified with 0.3% gellan gum; 10 replicates. After incubation in a plant growth incubator (25 °C, 80% relative humidity, 12-h photoperiod), the growth of seedlings roots was measured and photo-optically documented at 7 d. In addition, *B. plantarii*-free rice was cultivated in the greenhouse until the flowering stage, then the spikelets were sprayed-treated with zinc thiazole (0.05 mM) and *B. plantarii* (10^6^ CFU/mL), or *B. plantarii* (10^6^ CFU/mL) with water application as the negative control. At the maturation stage, samples from all treatments were subjected to quantification of tropolone secreted by *B. plantarii* [17].

### 2.7 Biological function of the rice-metabolized thiazole-class antimicrobial

Thiazole-class antimicrobials are metabolized into metal cations and thiazole in plants [36], thus tropolone secretion and cell growth of *B. plantarii* were systematically analyzed in response to zinc thiazole (a representative thiazole-class antimicrobial) and plant-transformed residues. *B. plantarii* was grown at 25 °C in the dark in MA media at 150 rpm with 0.005 mM, 0.05 mM and 0.5 mM zinc thiazole. At 0, 12, 24, 36, 48, 60, 72, 84 and 96-hours of cultivation, the optical density (OD) of media suspensions was measured at 660 nm. Simultaneously, tropolone was extracted and detected according to a previous report [17]. At the same concentration range, zinc sulfate (ZnSO_4_) and 5-Amino-1,3,4-thiadiazole-2-thiol (ATT) as the two metabolization products of zinc thiazole in plants were separately implemented to investigate their effects on *B. plantarii* growth and tropolone secretion.

### 2.8 Transcriptome analysis

To elucidate the mode of action by which ATT interferes with tropolone secretion in *B. plantarii*, transcriptome profiling was employed. Gene expression in *B. plantarii* was assessed upon exposure to thiazole below the MIC level and contrasted to a control without exposure to ATT. Total RNA of Bp was isolated and purified using the TRIzol method (Life Technologies, CA, USA) and sent to Personal Biotechnology Co., Ltd (Shanghai, China) for sequencing. Reads with remaining adapters and low quality sequences were filtered out. The resulting quality-filtered reads were subjected to mapping with a reference assembly accession (GCF_001411805.1) using Bowtie2 [37]. Read counts were determined with HTSeq 0.6.1p2 [38] and differentially expressed genes were identified with DESeq 1.18.0 (false discovery rate of 0.05 as threshold value) [39]. Network analysis and visualization were performed using Cytoscape 3.7.2 [40].

### 2.9 Gene knockout

To obtain specific mutants of *B. plantarii*, gene knockout was carried out according to a previous report [41]. Plasmid extraction and gel extraction were performed using the Omega Bio-tek DNA purification kit (ANNORON, Beijing, China) and specific restriction enzymes (TaKaRa, Shiga, Japan). Briefly, the upstream and downstream fragments of the target gene and the kanamycin resistance gene fragment amplified with pTnMod-Okm were fused to SacI/HindIII-digested pEX18Tc with the In-Fusion® HD Cloning Kit (TaKaRa, Shiga, Japan) to generate the pEX18Tc-trpE construct for gene knockout. The recombinant plasmid was transformed into *E. coli* WM3064 (auxotrophic to DAP) before conjugation with *B. plantarii*. The double-crossover recombinants of *B. plantarii* mutants were screened on LB agar plates containing 10% sucrose (w/v) and kanamycin. For verification of the double crossover PCR and sequencing analysis was included [42]. Detailed information related to the utilized primers is shown in Table S4.

### 2.10 Regulatory system interaction assays

Guided by the transcriptome profiling, interactions between Quorum Sensing (QS) and Two-component System (TCS) in *B. plantarii* were investigated by using a targeted assay series as described below. Briefly, *B. plantarii* was exposed to a QS inhibitor [43], carot-4-en-9,10-diol (at 20 and 100 mM), and expression of the representative QS (*plaI*) and TCS genes (*troK, troR1, troR2*) was analyzed by qRT-PCR with *rpoD* as a reference house-keeping gene. In addition, expression of TCS genes (*troK, troR1, troR2*) in *B. plantarii* wild type (WT) and *plaI* mutant (*ΔplaI*) were also analyzed. Moreover, QS genes expression and tropolone secretion in *B. plantarii* WT and *ΔtroK* was assessed. The detailed information for the utilized primers is shown in Table S4.

### 2.11 Effect of ATT on TroK function and virulence factor expression

To locate the molecular target of ATT, *B. plantarii* △*troK* was exposed to an ATT gradient at 0, 0.005, 0.05, 0.5 and 5 mM and tropolone secretion was analyzed at 72 h. In both, the wild type (WT) and *B. plantarii* △*troK*, tropolone secretion was comparatively analyzed in the ATT-treated group and control group (solvent only).

### 2.12 Confirmation of molecular interactions

To determine the binding affinity of different compounds to the identified molecular target in *B. plantarii*, a surface plasmon resonance (SPR) assay was carried out in a Biacore T200 instrument running in the multiple cycle kinetics mode. Purified TroK (Fig. S3) was immobilized onto a CM7 sensor chip via amine coupling as described in the manufacturer's protocol. Serial dilutions of ATT were obtained by using running buffer (10 mM PBS, pH 7.4, 150 mM NaCl) with an equal volume of 5% DMSO. The solutions were injected at a flow rate of 30 μl/min for 180 s, followed by dissociation for 300 s. To correct for the DMSO effect, solvent correction was applied before proceeding with the evaluation. The corrected response (reference-subtracted and solvent-corrected) for active surface vs. cycle number was obtained and the kinetic parameters were determined using the Biacore Evaluation Software 3.0.

### 2.13 Molecular docking

The structures of ATT and adenosine triphosphate (ATP) were transformed into 3D models, and their structural energy was minimized by using the respective protocol with a force field from CHARMm in the BIOVIA Discovery Studio 2016 (DS 2016; Accelrys) [44]. Based on the sequences of the TroK protein, different template proteins (Accession 4CTI) retrieved by BLAST searches (blastp) within Uniprot (https://www.uniprot.org) were used to construct the TroK structure. Homology modeling was performed using the built-in MODELER program on the basis of the target sequence aligned to the template sequences. The proposed binding sites were defined through protein cavities. ATT and ATP docking was conducted with the constructed protein model using the grid-based approach CDOCKER [45]. Within the CDOCKER results, the conformation of a ligand with the lowest CDOCKER Energy was selected as the probable binding conformation and the affinity between the receptor and the ligand was indicated by their CDOCKER Interaction Energy. In addition, SMART (http://smart.embl.de/) was performed for analysis of the TroK domain architecture [46].

### 2.14 Anti-virulence efficacy of ATT in rice plants grown in different geographical regions

Supervised field trials were carried out in three typical climatic zones of rice cultivation in China, including temperate zone (Heilongjiang, Jilin, Liaoning provinces), plateau zone (Yunan, Guizhou, Sichuan provinces) and subtropic zone (Jiangsu, Fujian, Zhejiang provinces). In each of the nine provinces, three rice paddies were randomly selected and included in a field trial with 20% zinc thiazole suspension concentrate (SC). In each paddy, 20% zinc thiazole SC was applied at 1,500 and 1,875 mL/10,000 m^2^, for three and four times during pre-harvesting stages respectively (a schematic diagram of the field trials is shown in Fig. S4). At the maturing stage, rice grains were collected from each field for simultaneous analysis of the average level of tropolone and ATT, and assessment of potential risk implications of ATT.

### 2.15 Risk assessments of ATT for consumers and beneficial members of the rice microbiota

To assess the dietary risk of ATT, available pesticide toxicological data was included in calculations of the hazard quotient (HQ), where the estimated daily intake (EDI) was divided by the relevant acceptable daily intake (ADI) as previously reported [2]. An unacceptable risk for chronic dietary intake of ATT is indicated if HQ>1, while the dietary risk is acceptable if HQ<1. In order to evaluate the potential effect of ATT on beneficial microorganisms associated with rice, we selected *Sphingomonas melonis* that was previously found to shape a disease-resistant rice phenotype as a representative bacterium [22]. *S. melonis* was plated on LB plates on which paper discs with a serial dilution of zinc thiazole were placed to screen for potential formation of halos indicating growth inhibition. In addition, *S. melonis* cultures (with/without ATT) were incubated on a shaker at 110 rpm at 25 °C in the dark and sampled at the exponential (36 h) and stationary phase (72 h), respectively. A potential impact of ATT supplementation on liquid cultures was evaluated by optical density measurements at a wavelength of 660 nm.

### 2.16 Statistical analysis

Statistical analysis was performed using ANOVA, Student-Newman-Keuls test and Student's t-test. Significance of differences between treatments was assessed using SPSS software (version 18.0). A canonical correspondence analysis (CCA) was performed to explore correlations between tropolone concentrations and different antimicrobials residues using Canoco 5.0 (Wageningen UR, Netherlands). The distribution of sampling sites was illustrated with ArcGIS 10.3.

### 2.17 Data availability

All raw sequence files were deposited in the Sequence Read Archive of NCBI. The *B. plantarii* genome was deposited under BioProject PRJNA323430 and transcriptome data were deposited under PRJNA534181.

## 3 Results and discussion

### 3.1 Virulence factor occurrence in field-grown rice correlates with the presence of specific agrochemicals

According to our preliminary survey (2012-2016), seven prevalent classes of antimicrobials are frequently detected in rice paddies in Zhejiang Province, China (Figs. S1 and S2). Tropolone concentrations, representing *B. plantarii's* main virulence factor, showed significant differences in rice plants grown in six regions of Zhejiang Province during long-term monitoring. Based on the preliminary data, representative fields in these regions were selected for systematical analyses of the relationship between *B. plantarii* virulence and frequently detected antimicrobials in rice.

As a result, tropolone levels in rice spikelets were found to follow a decreasing order: Z10 > Z9 > Z6 > Z7 > Z1 > Z4 (Fig. S1, Table S5). The highest concentration was observed at 0.097 mg/kg in Z10, while the lowest was only 0.009 mg/kg in Z4. We also found that the general detectability of antimicrobials in rice spikelets from the six regions was different (Table S5). When the level of each antimicrobial compound was compared between the contrasting regions Z10 and Z4, a higher level of kasugamycin, thifluzamide and thiazoles (thiazole-class antimicrobials) was found in the tropolone-low region Z4 (*p*<0.05), whereas a higher level of carbendazim, ethylicin, tebuconazole, tricyclazole, triadimefon, validamycin (*p*<0.05) was found in the tropolone-enriched region Z10 (Table S1), irrespective of comparable levels of trifloxystrobin.

**Fig. 1:**
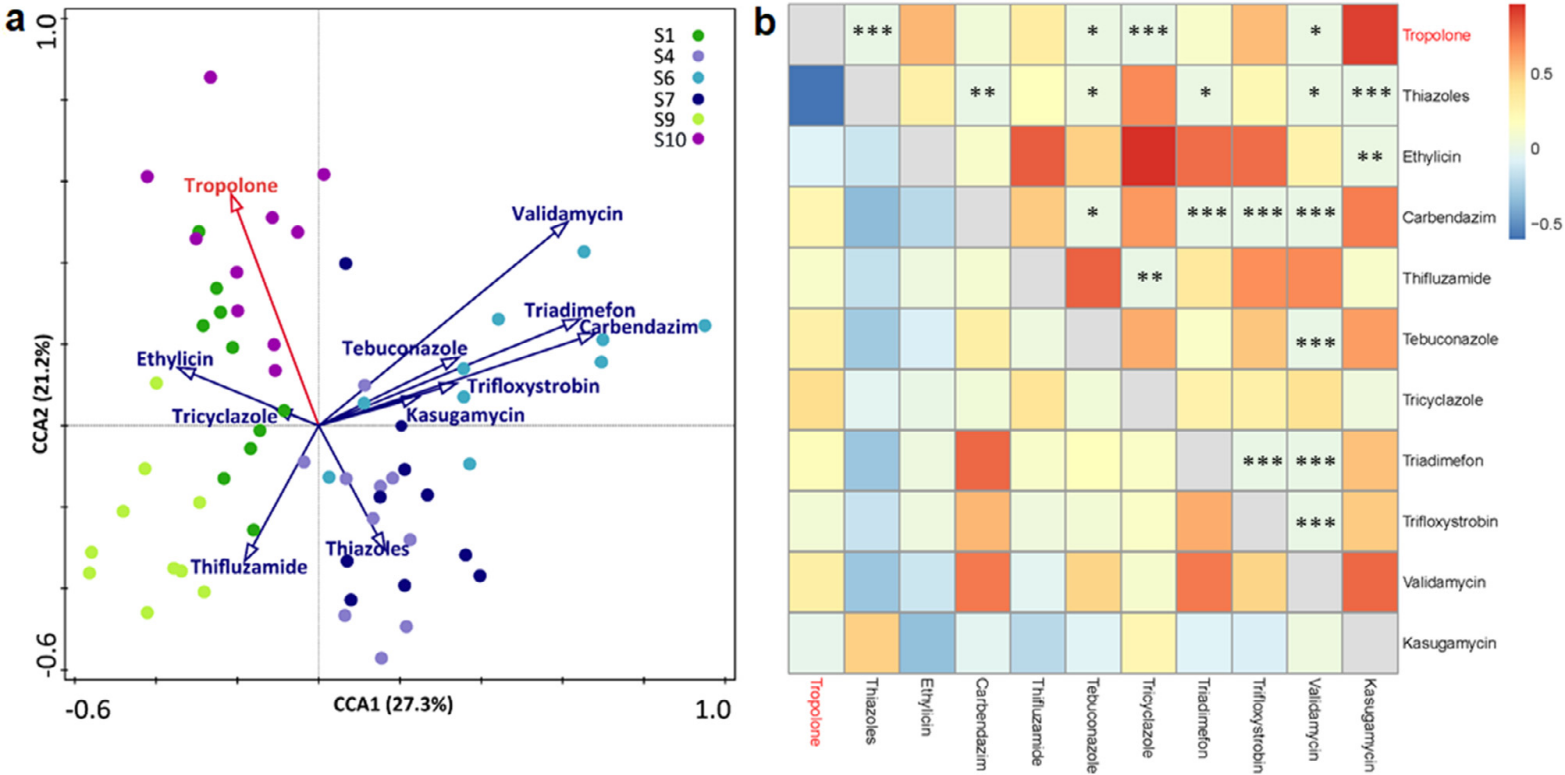
**Co-occurrence modeling of virulence factor expression and common antimicrobials in rice production.** The correlation between virulence factor expression (tropolone secretion by *B. plantarii*) and prevalent antimicrobials in rice cultivated in different production regions was visualized using canonical correspondence analysis (CCA) and a heat map with correlation coefficients. (a) In the CCA panel, the red arrow indicates the virulence factor tropolone, blue arrows indicate the antimicrobials. The colored circles represent different regions where rice is grown. (b) In the heat map with correlation coefficients, the regions above and below the gray diagonal line indicate the *P* value and *R* value, respectively. The color gradient in the heatmap indicates the relationship from positive (red) to negative (blue). *P < 0.05; **P < 0.01; ***P < 0.001.

A canonical correspondence analysis (CCA) was conducted to generate a co-occurrence model of the present antimicrobials and tropolone in rice. Here, CC1 and CC2 accounted for 48.5% of the total variance in the analyzed dataset (Fig. 1a). Different correlations were observed in the complex network, where most antimicrobials present in rice spikelets showed positive correlations with tropolone. Two triazoles, tebuconazole (*r* = 0.2955, *p*<0.05) and tricyclazole (*r* = 0.4428, *p*<0.005), as well as validamycin (*r* = 0.3154, *p*<0.05) showed a significant correlation (Fig. 1b). In contrast, thiazole showed a significant negative correlation with tropolone (*r* = -0.5996, *p*<0.005) (Fig. 1b).

### 3.2 Thiazole-class antimicrobial is a key modulator against virulence factor expression in *B. plantarii*

Most of the assessed antimicrobials and especially triazoles, were positively correlated with *B. plantarii* virulence (Fig. 1). This is most likely due to the mode of action of triazoles, which target the lanosterol-14α-demethylase (a cytochrome P450 enzyme) that is highly conserved in pathogenic fungi but not present in bacteria [47]. The conserved mode of action of triazole molecules might enhance the colonization and survival of *B. plantarii* in the plant's carposphere (micro-niche colonized by different microorganisms) by elimination of fungal competitors [48].

In contrast, antimicrobials with both fungicidal and bactericidal activities showed structure-specific effects on *B. plantarii*. For instance, the common glucosaminidase glycoside-class validamycin showed a significant positive correlation with tropolone secretion. This suggests that validamycin is not inhibiting *B. plantarii* at the prevailing levels in rice, which is in contrast to another important bacterial rice pathogen *Ralstonia solanacearum* (previously known as *Pseudomonas solanacearum*) [49]. The efficacy difference was confirmed with the conducted bioassays where the MIC for *B. plantarii* was over 2-fold higher than for *R. solanacearum* (Fig. S6). Validamycin shows high efficacy against the fungal pathogen *Rhizoctonia solani* Kühn-caused sheath blight disease and damping-off disease in various crops and vegetables [50]. The related mode of action was found to be based on inhibition of trehalase [51]. Few highly conserved regions were found in trehalases among the two bacteria and the fungus implemented in the current study (Fig. S7a). *R. solani* and *R. solanacearum* clustered in relatively close clades of the phylogenetic tree, while typical phytopathogenic strains of *B. plantarii* formed a separate clade (Fig. S7b). In combination with MIC assessments (Fig. S6), this indicates insufficient efficacy of validamycin to inhibit *B. plantarii*, but a high efficacy in terms of *R. solanacearum* and *R. solani* inhibition. More importantly, the finding provides indications that presence of validamycin in rice could pose a risk for increased *B. plantarii* virulence; the underlying mechanisms require further analysis.

**Fig. 2:**
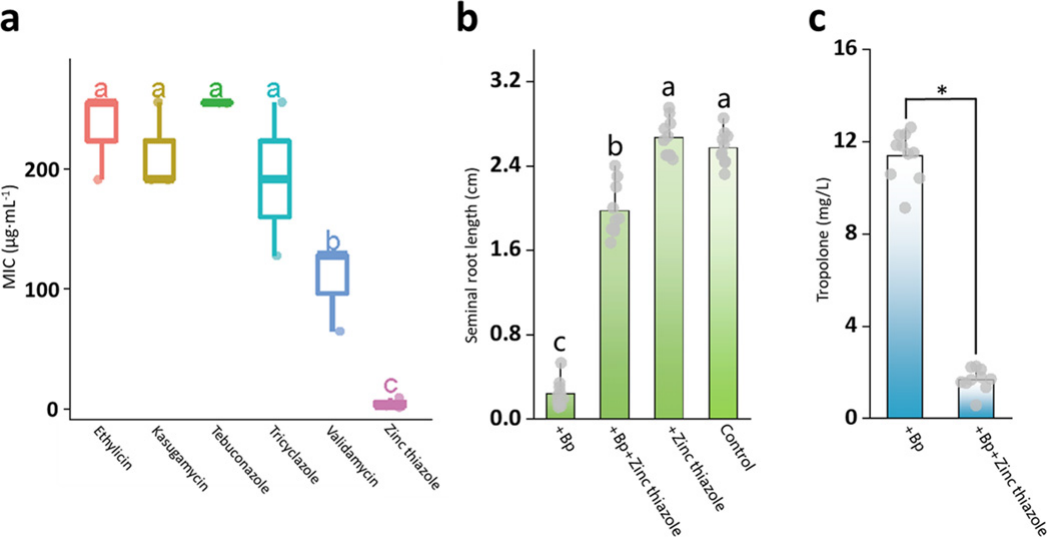
**Effect of different antimicrobials on *B. plantarii* growth and virulence factor expression *in vitro* and in the rice-pathosystem.** (a) The MICs of common antimicrobials against *B. plantarii* (Bp) were calculated based on an *in vitro* assay, values are means ± SD (shown as error bars, n = 3). (b) The growth performance of rice subjected to a combined treatment of Bp and/or the representative thiazole-class antimicrobial (zinc thiazole) was evaluated at the seedling stage, values are means ± SD (shown as error bars, n = 10). (c) Tropolone secretion by Bp in the rice spikelets was additionally quantified at the flowering stage, values are means ± SD (shown as error bars, n = 10). Letters indicate significant difference by Student-Newman-Keuls test (*P* < 0.005), * *P <* 0.001 by Student's-*t* test.

Thiazoles uniquely showed a negative effect on *B. plantarii* virulence factor expression in rice (Fig. 1), which is supported by its significant repression of *B. plantarii* population growth and the lowest MIC of the representative thiazole antimicrobial zinc thiazole, compared to the other representative antimicrobials detected in rice grains (Fig. 2a). Moreover, at the MIC level, zinc thiazole significantly reduced the development of disease development at the seedling stage of rice (Fig. 2b) and was shown to reduce virulence factor secretion of *B. plantarii* at the flowering stage (Fig. 2c). In addition, rice growth was not affected by its application in absence of *B. plantarii* (Fig. 2b). This implies that zinc thiazole synergistically exerts a strong effect in rice to inhibit *B. plantarii* virulence rather than by activation of the rice immune system. We preliminary hypothesized that a thiazole-class antimicrobial as a key modulator in the plant system plays a pivotal role in modulating *B. plantarii* virulence.

### 3.3 Inhibition of virulence factor expression by thiazole-class antimicrobial is not linked to antibacterial effects

Thiazole-class antimicrobials (thiazole moiety with various metal cations) have been widely applied in agricultural production since the past decade due to their efficacy against a broad spectrum of phytopathogens, but their mode of action remained so far unresolved [29,36,52]. For instance, thiazole-class antimicrobials are applied to control *Xanthomonas oryzae*-caused blight in rice production [29,53]. When applied to rice, the ATT anion and metal cation are transformed in the plant's above-ground tissues [36]. We have confirmed this observation at a later point in our study when we quantified thiazole in rice spikelets after different zinc thiazole treatments.

**Fig. 3:**
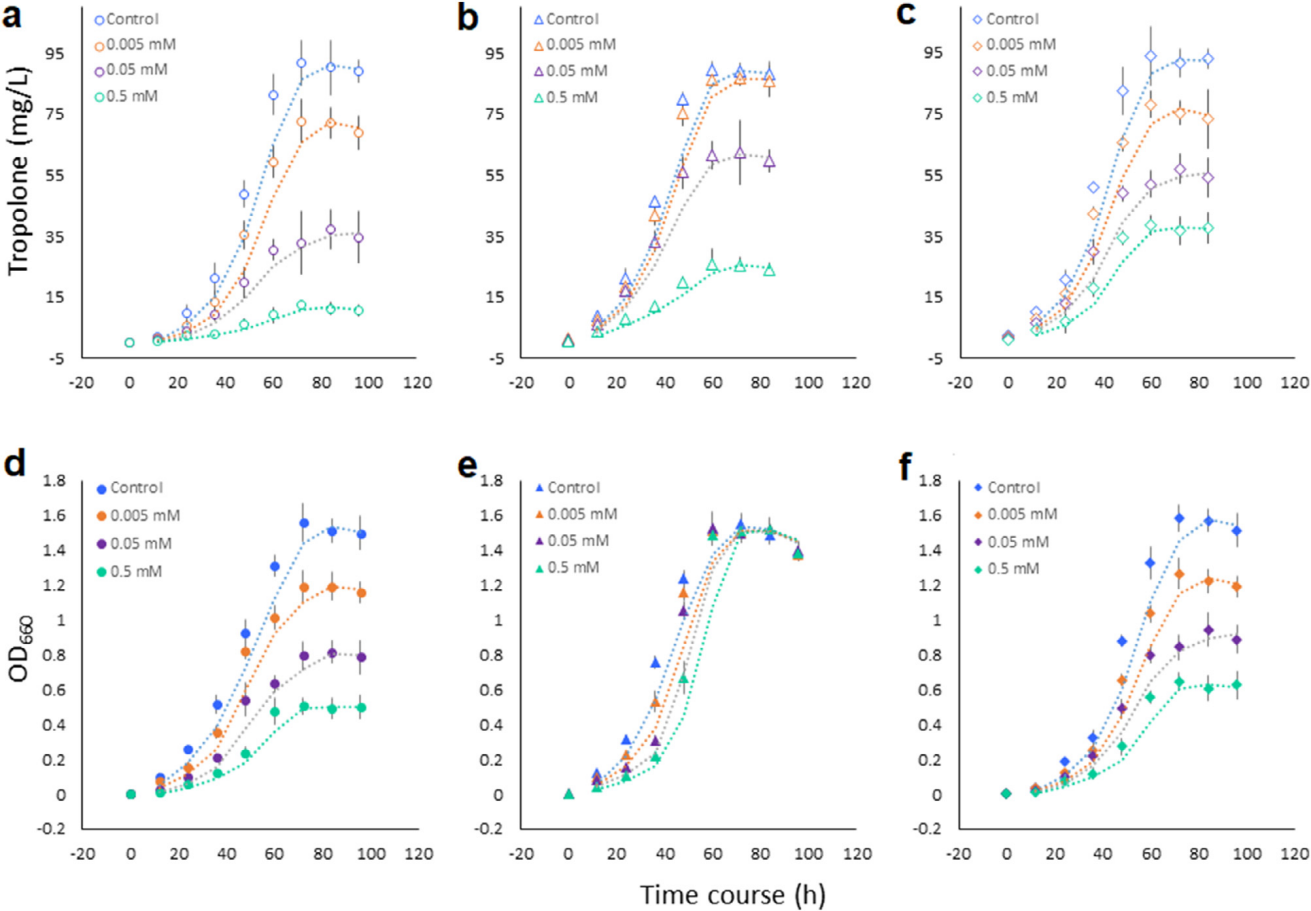
**Dynamics of cell growth and virulence factor expression of *B. plantarii* in response to the thiazole-class antimicrobial compound and its disassociated metabolites**. Dynamics of tropolone secretion (a-c) and cell growth (d-f) of *B. plantarii* were constantly observed at 0, 12, 24, 36, 48, 60, 72, 84 and 96-hours of cultivation upon exposure to diverse moieties of the thiazole-class antimicrobial compound. Zinc thiazole was selected as the representative thiazole-class antimicrobial and the treatment was divided into three moieties, including the parent molecule zinc thiazole (A&D); thiazole, the prevalent metabolite in rice (B&E) and the metal cation Zn^2+^ (C&F). Values are means ± SD (shown as error bars, n = 3).

We selected zinc thiazole as the model compound for deepening analyses and intended to clarify which structural moiety was involved in modulating *B. plantarii* virulence. Upon exposure to zinc thiazole (0.5 mM), tropolone secretion by *B. plantarii* was significantly repressed by 86.4% - 88.8% compared to blank controls obtained at 48 to 96 h, while supplementation of 0.005 mM and 0.05 mM zinc thiazole decreased the tropolone levels by 20.3% - 27.5% and 58.6% - 64.1% (Fig. 3a), respectively. In complementary treatments with the two constituents of zinc thiazole, Zn^2+^ and ATT (Fig. 3b, c), a similar pattern was observed with zinc sulfate (simulating application of Zn^2+^) where a dose-dependent reduction of tropolone was shown*.* The effects were different in case of ATT; significant reductions of tropolone secretion were only observed in the 0.5 mM and 0.05 mM treatments (Fig. 3b).

During the time course of cell growth (0-100 h), we found that both zinc thiazole and zinc sulfate dose-dependently inhibited cell growth (Fig. 3d, f), whereas ATT did not impact the cell growth of *B. plantarii* significantly (Fig. 3d, f). This implies that the decrease of tropolone secretion by *B. plantarii* exposed to zinc sulfate and zinc thiazole is attributable to the reduced cell density caused by the bactericidal activity of the zinc ion [54]. Interestingly, it suggests that the ATT moiety inhibits *B. plantarii* virulence factor expression via an unidentified regulatory effect, which we deemed to be of particular importance, owing the low risk of induction of AMR (antimicrobial resistance) [55].

### 3.4 ATT triggers transcriptome reprogramming in *B. plantarii*

To elucidate the mode of action by which ATT alleviated *B. plantarii* virulence, RNA-seq and transcriptome profiling were employed to investigate differences in gene expression in response to ATT exposure. The *r* value ranged from 0.971-0.998 in the RNA-seq datasets (Fig. S8a), indicating a robust repeatability of the obtained data for further analysis. Upon exposure to ATT, we found differences in the expression of 2050 genes (Fig. S8b), among which 585 genes were significantly up-regulated (Dataset S1; log2-transformed fold change>1, *p*<0.05), while 1465 genes were significantly down-regulated (Dataset S2).

**Fig. 4:**
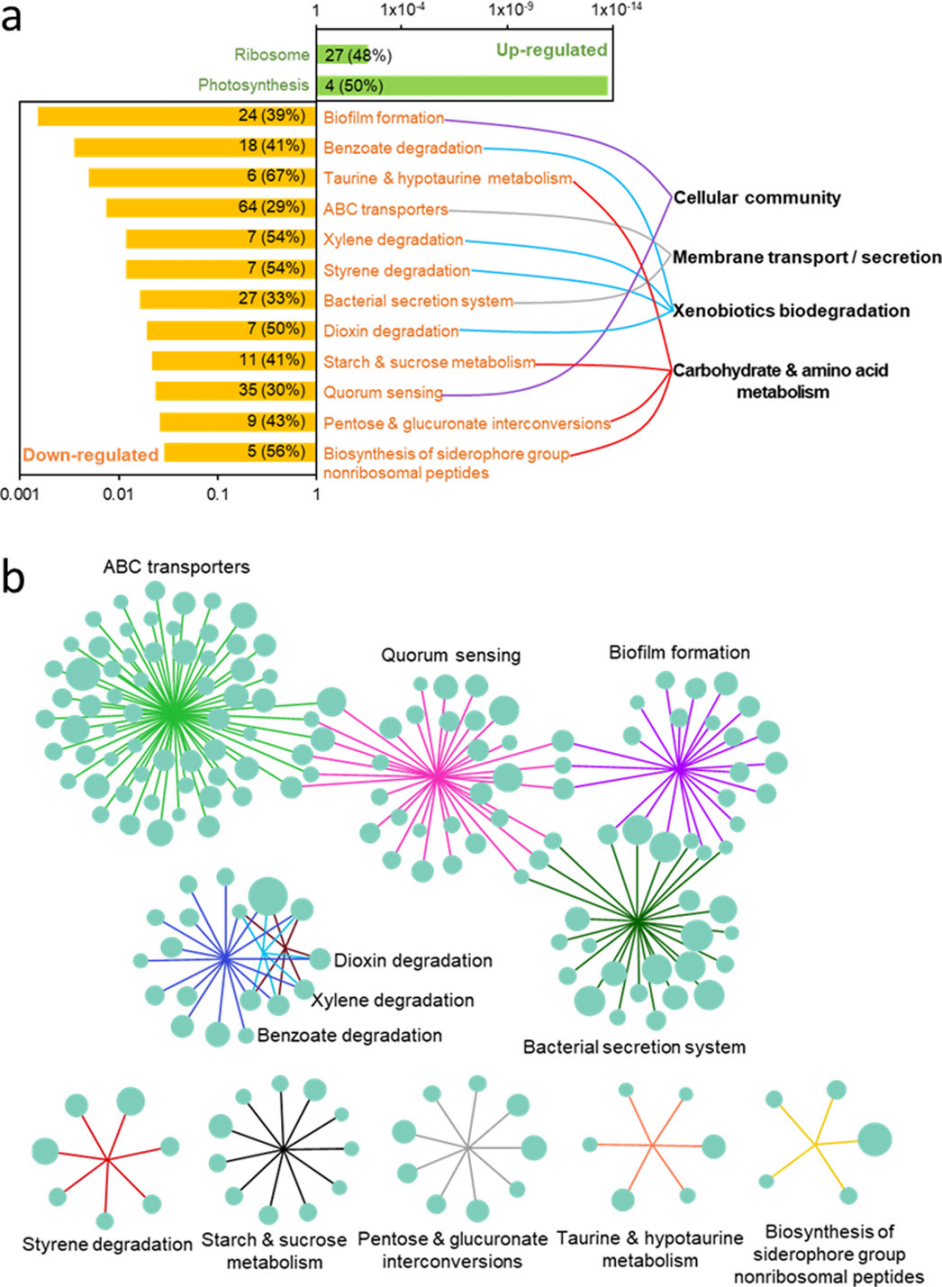
**Network analysis of transcriptome-level alterations in *B. plantarii* as a response to thiazole exposure.** (a) Enriched KEGG pathways with significantly up- and down-regulated genes. The x-axis indicates P values (the distance is log10-transformed) of KEGG pathway enrichments. The numbers in the plot show the number of differentially expressed genes in each enriched pathway, with the percentage (in parentheses) of all genes in the corresponding pathways. The enriched pathways with significantly down-regulated genes were further clustered in four high-level pathways/processes, which are shown by curves in different colors. (b) The relationship between significantly down-regulated genes and the corresponding enriched KEGG pathways. Each gene is indicated with a green circle and the relative fold-change (log2-transformed) of gene expression is indicated by circle size. Different pathways of these genes are indicated with lines in different colors.

KEGG pathway enrichment analysis indicated that only two pathways including ribosome and photosynthesis can be reconstructed from significantly up-regulated genes (Fig. 4a, Dataset S3; *p*<0.05). In contrast, twelve KEGG pathways were enriched as indicated by down-regulated genes, which can be divided into four KEGG orthologies including cellular community, membrane transport/secretion, xenobiotics biodegradation, and carbohydrate and amino acid metabolism (Fig. 4a, Dataset S4; *p*<0.05).

To further investigate the pathways that are substantially impacted in response to ATT, a network showing relationships between the enriched genes and their involved pathways was constructed. We observed that four pathways including ABC transporters, QS, biofilm formation and Bacterial Secretion Systems (BSS) were connected closely and formed a primary cluster, while the three degradation pathways involving dioxin, xylene and benzoate formed a secondary cluster (Fig. 4B), which was followed by five individual pathway clusters.

Previous studies focusing on thiazole-class antimicrobials were conducted with *Xanthomonas oryaze* pv. *oryzae*, in which zinc thiazole and bismerthiazol (an ATT analog) were found to promote rice defense against the pathogenic *Xanthomonas* via inhibition of extracellular polysaccharides (EPS) that is an essential component for biofilm formation [29]. Interestingly, biofilm formation similar to other virulence factors are known to be under control of QS in most bacteria [56], [57], [58]. By comparatively assessing the available information, we found indications for the mode of action of thiazole in *B. plantarii* in the transcriptome (Fig. 4). These together suggested that attenuated virulence of *B. plantarii* in response to ATT was associated with disrupted QS-centered signaling cascades.

### 3.5 ATT-triggered transcriptome reprograming in *B. plantarii* is mediated via TCS

Also, we noticed that BSS of *B. plantarii* were remarkably enriched in the ATT-triggered transcriptome reprogramming, in which 27 genes including type II, type III and typeVI secretion systems were significantly down-regulated by at least 2.071-fold (Fig. S9, Dataset S2). Interestingly, BSS is known as a dominant downstream signaling pathway to be positively regulated by TCS and considered as a marker gene of TCS signal transduction. For instance, in *Pseudomonas syringae*, TCS RhpR/S is reported to regulate the type III secretion system [59], while TCS PhoB/R together with the ferric uptake regulator sense phosphate and iron to control virulence genes in type III and VI secretion systems in *Edwardsiella tarda* [60].

In *B. plantarii*, QS and TCS were both identified to control tropolone irrespective of their relationship [61,62]. We further subjected *B. plantarii* to a QS inhibitor previously identified, carot-4-en-9,10-diol, and found that expression of the QS marker gene (*plaI*) was down-regulated, without significant alteration in TCS gene (*troK, troR1* and *troR2*) expression (Fig. S10). Moreover, there was no difference in expression of *troK, troR1* and *troR2* between the wild type (WT) and *plaI* mutant (*ΔplaI*) (Fig. S11), which suggested that QS is not involved in regulating TCS gene expression. Reversely, down-regulated expression of the QS marker gene along with inhibited tropolone secretion was observed in the *troK* mutant (*ΔtroK*) in contrast to the WT of *B. plantarii* (Fig. S12). TCS functioning in *B. plantarii* was similar to those previously described in most Gram-negative bacteria, typically *Burkholderia, Pseudomonas* and *Vibrio*, where QS is modulated by different members of the TCS system [63], [64], [65]. This also suggested that ATT-triggered reprograming of the QS-centered signaling cascade (Fig. 4) and the subsequent attenuated virulence (Fig. 3) was likely to be mediated by TCS in *B. plantarii*.

### 3.6 ATT binds to a histidine kinase regulator required for virulence factor expression

**Fig. 5:**
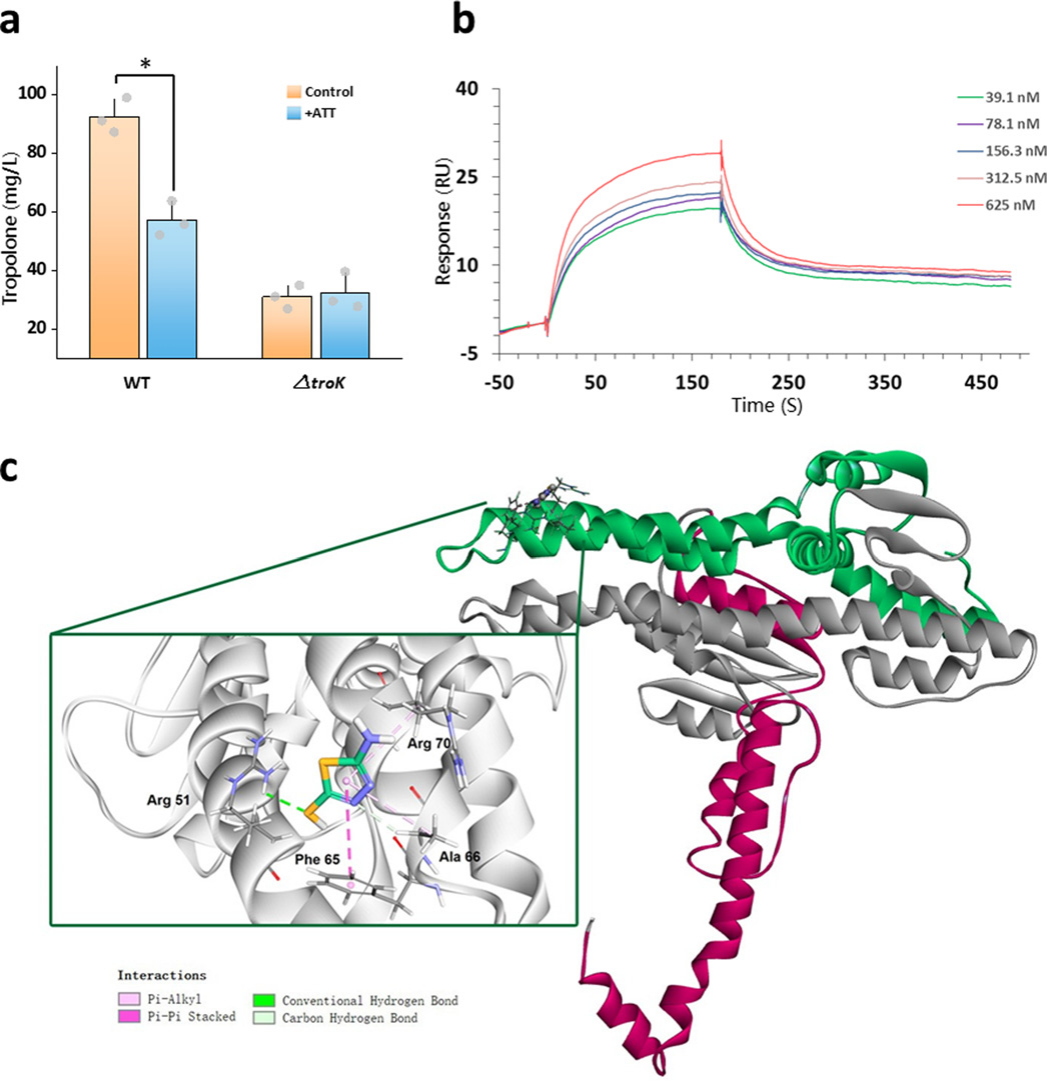
**Molecular insights into anti-virulence effects of thiazole on *B. plantarii*.** (a) Effect of thiazole on tropolone secretion in *B. plantarii* of wild type (WT) and TroK mutant (△*troK*). (b) SPR analysis of thiazole binding to TroK. Kinetic sensorgrams of the binding to TroK were obtained with serial dilutions. The responses were reference-subtracted, solvent-corrected and blank-deducted. (c) Molecular docking for binding sites of interaction between thiazole and amino acid residues of TroK. Letters indicate significant difference by Student-Newman-Keuls test (*P* < 0.005), * *P <* 0.001 by Student's-*t* test. Values are means ± SD (shown as error bars, n = 3).

Surprisingly, within non-significantly enriched KEGG pathways (*p*>0.05), 36 TCS genes were significantly altered upon exposure to ATT (Fig. S13, Dataset S2). A large portion of histidine kinase-coding genes including *tctE, rcsC, cusC* and *kdpD* were significantly down-regulated by at least 2.063-fold, while tropolone-controlling genes *troK, troR1* and *troR2* remained unaffected (Dataset S2). During the subsequent screening of knockout mutants, we found no difference in tropolone secretion between the ATT-treated and ATT-free group in the *ΔtroK* mutant, despite that they were both drastically repressed compared to the WT under the thiazole-free condition (Fig. 5a).

TroK is a class of histidine protein kinase (HPK), of which subsequent auto-kinase activity is generally dependent on an environmental condition, but the dose-dependent antagonism by thiazole was erased in *ΔtroK* (Fig. S14). This implied that thiazole was likely to bind to TroK directly to exert the antagonism. Using surface plasmon resonance (SPR) and complementary molecular docking, we confirmed that thiazole could directly bind to the purified TroK in the transmembrane region (Figs. 5b, c and S15) with target affinity [KD (equilibrium dissociation constant) value] of 0.728 μM (Table S6), which possibly led to impairment of normal configuration required for intracellular ATP-TroK binding at the HATPase_c pocket (Figs. S15, S16). However, the detailed mechanism underpinning blocked auto-phosphorylation requires further investigation.

### 3.7 Anti-virulence effect of ATT across different climatic regions and its effects on beneficial microbes

**Fig. 6:**
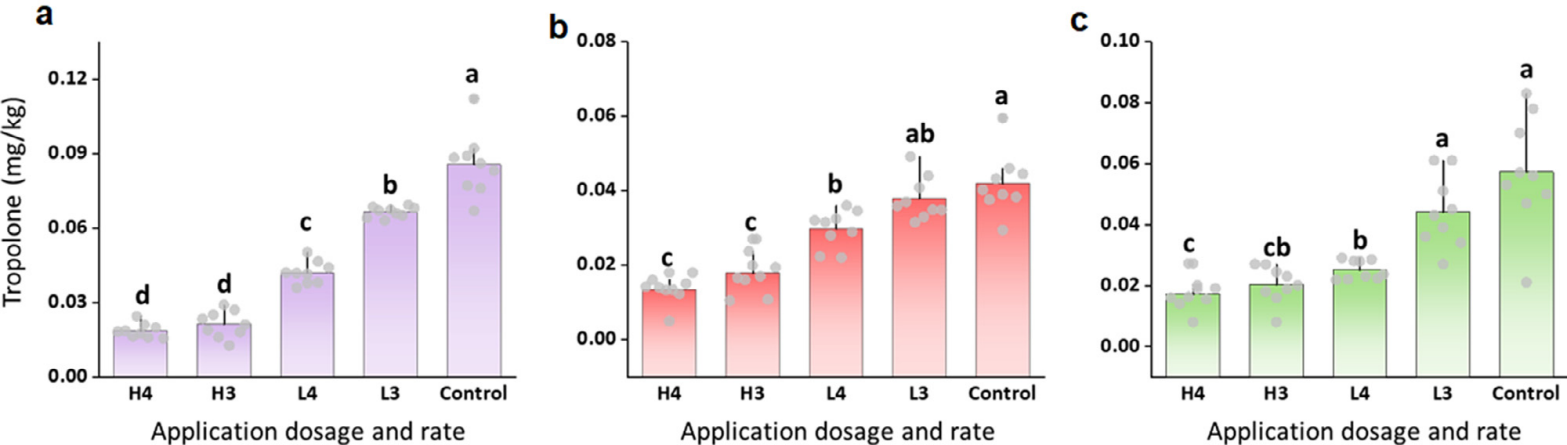
**Anti-virulence efficacy of thiazole in rice cultivated in different geographical regions.** Three climatic zones representative for rice cultivation in China including a temperate zone (a), a subtropic zone (b) and a plateau zone (c) were implemented. Zinc thiazole (20% SC) was selected as the representative thiazole-class antimicrobial to test anti-virulence effect of thiazole in geographically separated rice fields. Applications were conducted at a low (L) dosage (1,500 mL/10,000 m^2^) and a high (H) dosage (1,875 mL/10,000 m^2^) with the numbers indicating either three or four consecutive applications during the growth stage of rice plants. Letters indicate significant difference by Student-Newman-Keuls test (*P* < 0.05). Values are means ± SD (shown as error bars, n = 10).

Virulence factor expression by *B. plantarii* in rice is largely dependent on the overwintered pathogen population in the environmental epidemics cycle, which implies that geo-climatic factors are involved. Therefore, the anti-virulence effect of ATT during rice cultivation was confirmed in three typical climatic zones in China that were previously connected with *B. plantarii* occurrence [17]. Tropolone levels ranged from 0.041 to 0.085 mg/kg in the control plots, whereas 0.013 to 0.021 mg/kg were found in the plots treated with a high dosage of 20% zinc thiazole suspension concentrate (SC), and concentrations from 0.025 to 0.066 mg/kg in those treated with a low dosage of 20% zinc thiazole SC (Fig. 6). Tropolone was decreased up to 68.3%, 70.2% and 78.8% in the zinc thiazole-treated plots in contrast to the control plots in subtropic, plateau and temperate zones, respectively (Fig. 6). This evidenced that the anti-virulence effects of ATT on *B. plantarii* can be reproduced in representative agroecosystems across different climatic regions.

The level of ATT was also assessed in matured rice grains. It ranged between 0.0157-0.0654 mg/kg and 0.109-0.109 mg/kg, when 20% zinc thiazole SC was sprayed either three or four times at 1,500 and 1,875 mL/10,000 m^2^, respectively (Fig. S17). The HQ for zinc thiazole in rice was calculated as 0.043, indicating that long-term consumption of rice products containing ATT would not pose unacceptable dietary risks towards the general population. Moreover, ATT did not show significant inhibition towards resident beneficial microbes of rice (Fig. S18). Taken together, our results highlight that ATT is a promising agent for the development of upcoming plant protection strategies.

## 4 Perspectives and conclusion

Previously, targeted assessments uncovered why rainfalls or a period of high humidity are crucial environmental factors modulating pathogen-plant interactions resulting in disease outbreaks [66]. In contrast, anthropogenic and environmental factors that interfere with pathogen-plant interactions in agroecosystems could provide a promising basis for disease prevention, yet they remained largely unexplored [25]. Guided by the evident variation in pathogen virulence factor expression observed in rice grown in different and geographically distant locations, we deemed that this unusual phenomenon might reflect varying natural resilience against pathogens in local agroecosystems as previously described [67]. By following an investigative approach, a ubiquitous thiazole-class compound enriched by agrochemical treatments in rice was discovered as a key factor that substantially lowered virulence during pathogen infection. Deepening assessments have confirmed a modulatory role that is replicable irrespective of the geographic location and climate zone. Our findings imply that anthropogenic factors, especially those that have potentially negative effects on the virulence of phytopathogens towards host plants, should be explored in prevalent agroecosystems for a better understanding of the “disease triangle” paradigm [24].

From the classical perspective, the metabolic capacity of plants has been negatively associated with *in situ* antimicrobial efficacy of agrochemicals against pathogens*.* In the present study, an unexpected, and at least from the human perspective positive, off-target effect exerted by a non-antimicrobial thiazole-class compound that originated from a plant metabolized-antimicrobial was observed. Metabolization in the plant led to the accumulation of the non-antimicrobial metabolite, which reduces the pressure for selecting AMR-equipped pathogens, a prevalent concern in agroecosystems that are frequently exposed to antimicrobials [68,69]. The anti-virulence effect triggered by the plant-converted agrochemical has substantial implications for supporting the plant's defense system in counteracting bacterial pathogens armed with small molecule-based virulence factors. This more profoundly implies that the plant's metabolic system in combination with exogenous chemicals might play a synergistic role in resisting pathogen-meditated biotic stress.

Moreover, we provide insights at the molecular level into the anti-virulence efficacy of ATT, which was attributable to its TroK histidine kinase-antagonizing activity, leading to interference with the down-stream signaling cascade required for virulence factor expression. As a matter of fact, the histidine kinase family plays an essential role in sensing host plants, thus controlling the virulence in various bacterial phytopathogens [70]. Our finding thus suggests that novel, environmentally safe histidine kinase-targeting agents might be a promising strategy to control bacterial phytopathogens epidemics globally.

Collectively, we have uncovered a so far unknown biological role of a metabolized metallo-thiazole as an anti-virulence modulator in a host plant to counteract bacterial pathogen infection in phyllosphere. Modulation of histidine kinase activity, virulence factor expression, and other physiological processes by naturally occurring thiazole analogs should be further investigated as they may prove to be present in other plant-pathogen interactions emerging in various agro-ecosystems.

## Declaration of Competing Interests

The authors have declared no conflicts of interests in this work.

## Appendix Supplementary materials

Image, application 1

Image, application 2

Image, application 3

Image, application 4

Image, application 5
