# Supplementary material for: Reprogramming of phytopathogen transcriptome by a non-bactericidal pesticide residue alleviates its virulence in rice
Source: Fundam Res. 2022 Jan 20;2(2):198–207. doi: 10.1016/j.fmre.2021.12.012 (PMC11197535; doi:10.1016/j.fmre.2021.12.012)
Supplement: Supplementary file 1 [file mmc1.docx]

**Supporting Information**

**Reprogramming of phytopathogen transcriptome by a non-bactericidal pesticide residue alleviates its virulence in rice**

Haruna Matsumoto ^a,b^, Yuan Qian ^a^, Xiaoyan Fan ^a^, Sunlu Chen ^e^, Yanxia Nie ^f^, Kun Qiao ^a^, Dandan Xiang ^a,g^, Xinzhong Zhang ^h^, Meng Li ^a^, Bo Guo ^i^, Peilin Shen ^a,j^, Qiangwei Wang ^a,b^, Yunlong Yu ^a,b^, Tomislav Cernava ^c,^ *, Mengcen Wang ^a,b,d,^ *

1. Key Laboratory of Molecular Biology of Crop Pathogens and Insects, Ministry of Agriculture, Institute of Pesticide and Environmental Toxicology, Zhejiang University, Hangzhou 310058, China
2. Key Laboratory of Biology of Crop Pathogens and Insects of Zhejiang Province, College of Agriculture and Biotechnology, Zhejiang University, Hangzhou, China
3. Institute of Environmental Biotechnology, Graz University of Technology, Petersgasse 12, 8010 Graz, Austria
4. Global Education Program for AgriScience Frontiers, Graduate School of Agriculture, Hokkaido University, Sapporo 060-8589, Japan
5. State Key Laboratory of Crop Genetics and Germplasm Enhancement, Nanjing Agricultural University, Nanjing 210095, China
6. Ecology and Environmental Sciences Center, South China Botanical Garden, Chinese Academy of Sciences, Guangzhou 510650, China
7. Key laboratory of South Subtropical Fruit Biology and Genetic Resource Utilization (MOA), Institute of Fruit Tree Research, Guangdong Academy of Agricultural Sciences, Guangzhou 510640, China
8. Tea Research Institute, Chinese Academy of Agricultural Sciences, Hangzhou 310008, China
9. Shanghai International Studies University, Shanghai 200083, China
10. Xiaoshan Agricultural Comprehensive Development Zone & Management Committee, Hangzhou 311200, China

* Co-corresponding author

To whom correspondence should be addressed: wmctz@zju.edu.cn

**Keywords:** Phytopathogen, virulence factor, transcriptome reprogramming, agrochemical, pesticide, rice

Table S1. UPLC conditions for quantification of thiazoles.

| Time/ min | Flow rate (mL/min) | Mobile phase | |
| --- | --- | --- | --- |
|  |  | 0.1% Formic acid (%) | Acetonitrile  (%) |
| 0.00 | 0.30 | 60 | 40 |
| 2.00 | 0.30 | 20 | 80 |
| 3.00 | 0.30 | 80 | 20 |

Note: Thiazoles were represented by 5-Amino-1,3,4-thiadiazole-2-thiol, which is a ubiquitous metabolite of all thiazole-class antimicrobials in plants.

Table S2. MS/MS parameters for quantification of thiazoles under MRM mode.

| Analyte | Retention time | Qualifier (m/z) | Quantifier (m/z) | | Cone (V) | CE (eV) |
| --- | --- | --- | --- | --- | --- | --- |
| Thiazole | 1.00 | 134.09/43.19 | 134.09/75.06 | 40 | | 20 |
|  |  | 134.09/75.06 |  | 40 | | 25 |

Note: MRM, Multi Reaction Monitoring. Thiazoles were represented by 5-Amino-1,3,4-thiadiazole-2-thiol, which is a ubiquitous metabolite of all thiazole-class antimicrobials in plants.

Table S3. Recovery of representative antimicrobials in rice spikelets (n = 10).

| Antimicrobials | Spiked level  (mg/kg) | Recovery  (%) | RSD  (%) |
| --- | --- | --- | --- |
| Thiazoles | 0.01 | 79 | 12.5 |
|  | 0.1 | 88 | 5.2 |
|  | 1.0 | 92 | 8.9 |
| Ethylicin | 0.01 | 82 | 7.1 |
|  | 0.1 | 99 | 5.4 |
|  | 1.0 | 87 | 10.9 |
| Carbendazim | 0.005 | 88 | 13.0 |
|  | 0.05 | 90 | 3.1 |
|  | 0.5 | 91 | 2.0 |
| Thifluzamide | 0.001 | 76 | 6.7 |
|  | 0.01 | 85 | 0.8 |
|  | 0.1 | 102 | 1.4 |
| Tebuconazole | 0.001 | 75 | 8.2 |
|  | 0.01 | 88 | 3.7 |
|  | 1.0 | 94 | 2.6 |
| Tricyclazole | 0.005 | 83 | 1.0 |
|  | 0.05 | 90 | 5.5 |
|  | 0.5 | 92 | 6.4 |
| Triadimefon | 0.005 | 88 | 1.2 |
|  | 0.05 | 84 | 3.6 |
|  | 0.5 | 107 | 2.3 |
| Trifloxystrobin | 0.05 | 85 | 2.4 |
|  | 0.5 | 112 | 6.8 |
|  | 5.0 | 99 | 4.3 |
| Validamycin | 0.001 | 69 | 2.0 |
|  | 0.01 | 76 | 1.3 |
|  | 0.1 | 79 | 1.7 |
| Kasugamycin | 0.004 | 72 | 3.5 |
|  | 0.04 | 75 | 2.6 |
|  | 0.4 | 80 | 1.4 |

Note: Thiazoles were represented by 5-Amino-1,3,4-thiadiazole-2-thiol, which is a ubiquitous metabolite of all thiazole-class antimicrobials in plants.

Table S4. Oligonucleotide primers used in this work.

| Gene/  Primer names | Forward primer (5’-…-3’) | Reverse primer (5’-…-3’) | Purpose/function |
| --- | --- | --- | --- |
| *treBp* | CGTAGTCGCCGTAATAGC | GAGAAGACCATCGTGAAGG | Identification of trehalases gene of *B. plantarii* |
| *treRa* | TGATCTGGTAGGCGAAGT | GCTCTATGGCGAACTGTT | Identification of trehalases gene of *R. solanacearum* |
| *treRh* | GCAGTCTAGCGATGAGTG | AGGAAGCGATAGGCAAGA | Identification of trehalases gene of *R. solani* |
| *troK* | AAGTAGATCATCTGCCAGTC | GGTGATGTTCTCGCTGTC | Quantification of TCS genes of *B. plantarii* |
| *troR1* | GTCGTATTCGTCGCTGAG | GAATGAAATCGTCGGAGAAC | Quantification of TCS genes of *B. plantarii* |
| *troR2* | TCACCTGATAGCCGTAGC | GCAGGACAAGGAGGATGT | Quantification of TCS genes of *B. plantarii* |
| *plaI* | ATGCAGACTTCCGTTCAC | AACACGCGGTAACGATAG | Quantification of QS genes of *B. plantarii* |
| *rpoD* | GCATCTTGTCGAACCACT | ACGAGGACGAAGAGGAAG | House-keeping gene in *B. plantarii* |
| *Kan* | TGTCTCAAAATCTCTGATGTTAC | TTAGAAAAACTCATCGAGCATC | Amplification of kanamycin resistance gene from pTnMod-Okm for gene knockout |
| pEX18Tc (v) | GCACGACAGGTTTCCCGACTG | CCGCTTCTGCGTTCTGATTTA | Verification of the pEX18Tc-related constructions |
| *plaI*-up | ATGATTACGAATTCGCTGCTGCTCGTTTTCCGCCG | GATGAGTTTTTCTAAGGTGTCCTCGTGGTAATTCC | Amplification of upstream fragment for *plaI* knockout |
| *plaI*-down | AGAGATTTTGAGACAGTTGCACGGGCGGCGCCGG | GGCCAGTGCCAAGCTCGCGTGATCGACGCCAGTTC | Amplification of downstream fragment for *plaI* knockout |
| *plaI* | CTGGGCTCCTATCGTTACC | AGCACGTACACCGTATCG | Verification of the *B. plantarii △plaI* by PCR or sequencing |
| *troK*-up | ATGATTACGAATTCGTCATGCCAGCGTCCTCGG | AGAGATTTTGAGACACGAGCCTGGTCTCGCGCG | Amplification of upstream fragment for *troK* knockout |
| *troK*-down | AGAGATTTTGAGACAGTTGATAATCGCCTCGCGA | GGCCAGTGCCAAGCTGTGACTGAACCTGCAAGC | Amplification of downstream fragment for *troK* knockout |
| *troK* | GCCGTAGTAGTTGGTGTG | GGTGATGTTCTCGCTGTC | Verification of the *B. plantarii △troK* by PCR or sequencing |

Note: 15 bp overlap (5’) in the primers’ sequences for in-fusion cloning is underlined.

Table S5. Level of antimicrobials and virulence factor expression in rice spikelets collected from six regions in Zhejiang province (n = 10).

| Location | Carbendazim  (mg/kg) | Ethylicin  (mg/kg) | Kasugamycin  (mg/kg) | Thifluzamide  (mg/kg) | Tebuconazole  (mg/kg) | Tricyclazole  (mg/kg) | Triadimefon  (mg/kg) | Trifloxystrobin  (mg/kg) | Validamycin  (mg/kg) | Thiazoles  (mg/kg) | Tropolone  (mg/kg) |
| --- | --- | --- | --- | --- | --- | --- | --- | --- | --- | --- | --- |
| Z1 | 0.008±0.006 | 0.028±0.009 | 0.007±0.006 | 0.001±0.001 | 0.007±0.006 | 0.006±0.003 | 0.009±0.008 | 0.05^a^ | 0.007±0.005 | 0.087±0.021 | 0.018±0.005 |
| Z4 | 0.014±0.008 | 0.011±0.002 | 0.037±0.017 | 0.009±0.008 | 0.010±0.013 | 0.036±0.009 | 0.017±0.008 | 0.05^a^ | 0.004±0.005 | 0.208±0.089 | 0.009±0.003 |
| Z6 | 0.077±0.031 | 0.018±0.010 | 0.014±0.007 | 0.013±0.009 | 0.119±0.099 | 0.034±0.008 | 0.219±0.125 | 0.0647±0.020 | 0.047±0.013 | 0.035±0.018 | 0.047±0.007 |
| Z7 | 0.035±0.012 | 0.010^a^ | 0.008±0.006 | 0.002±0.001 | 0.058±0.030 | 0.008±0.004 | 0.049±0.018 | 0.05^a^ | 0.014±0.009 | 0.053±0.019 | 0.024±0.005 |
| Z9 | 0.015±0.010 | 0.025±0.015 | 0.004^a^ | 0.024±0.013 | 0.041±0.022 | 0.043±0.019 | 0.028±0.009 | 0.052±0.006 | 0.001^a^ | 0.027±0.008 | 0.050±0.006 |
| Z10 | 0.031±0.011 | 0.012±0.003 | 0.024±0.011 | 0.005±0.008 | 0.064±0.035 | 0.044±0.026 | 0.048±0.018 | 0.05^a^ | 0.022±0.0156 | 0.014±0.005 | 0.097±0.011 |

Note: Superscript “a” indicates that the detectable level was lower than LOQ of the analytes. Virulence factor expression in *B. plantarii* was assessed by quantification of the tropolone level. Thiazoles were represented by 5-Amino-1,3,4-thiadiazole-2-thiol, which is a ubiquitous metabolization product of all thiazole-class antimicrobials in plants.

Table S6. Kinetic and thermodynamic constants of ATP and ATT during binding to TroK.

| Kinetic and thermodynamic constants | | Thiazole | ATP |
| --- | --- | --- | --- |
| Kinetic constants | K_a_ (M^-1^·s^-1^) | 9.47E+3 | 2.87E+4 |
|  | K_d_ (s^-1^) | 6.89E-3 | 3.23E-3 |
| Thermodynamic constants | K_D_ (M) | 7.28E-7 | 1.13E-7 |

Note: K_a_, Kinetic association constant; K_d_, Kinetic dissociation constant; K_D_, Thermodynamic dissociation constant (affinity constant); ATT, 5-Amino-1,3,4-thiadiazole-2-thiol.


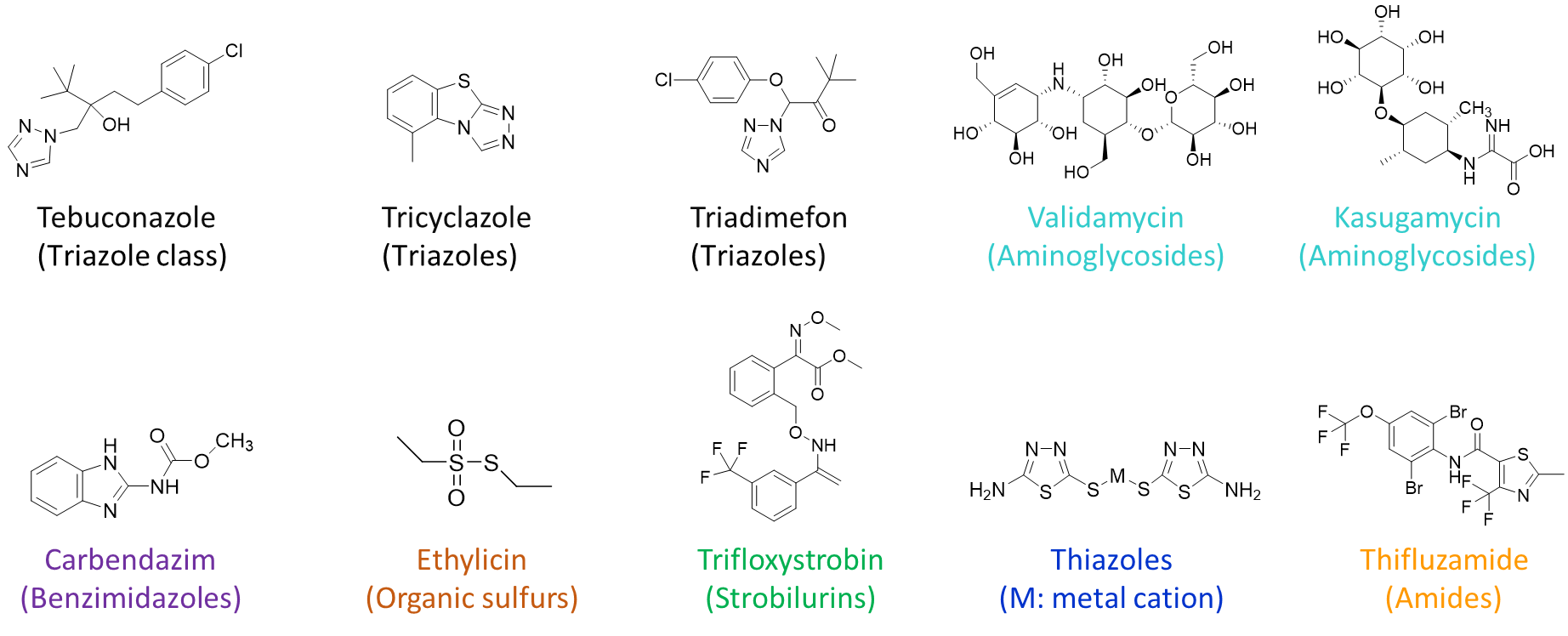


Figure S1. Typical chemical structures of seven classes of antimicrobials used in rice farming. Different colors indicate different chemical classes. Thiazoles indicates thiazole-class antimicrobials that are applied in rice cultivation.


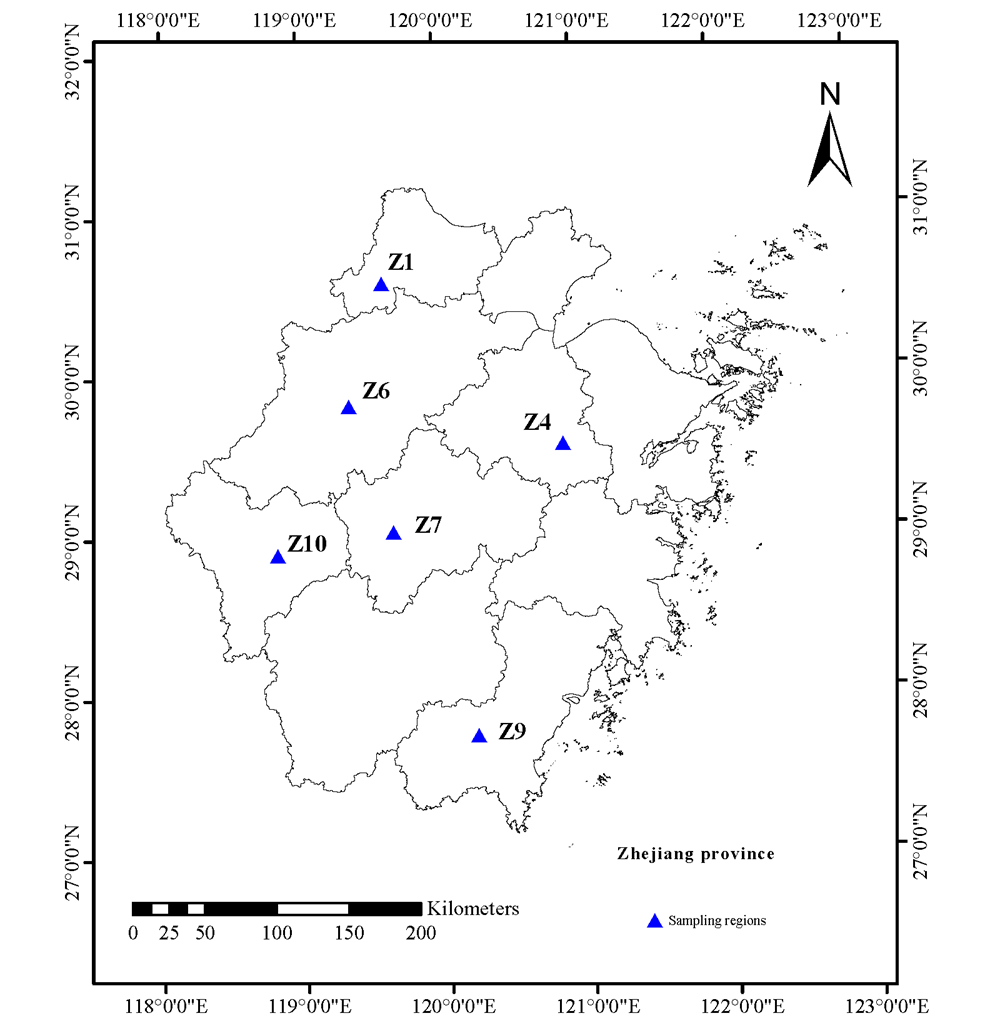


Figure S2. Location of six representative regions that were implemented for rice sampling in Zhejiang Province.


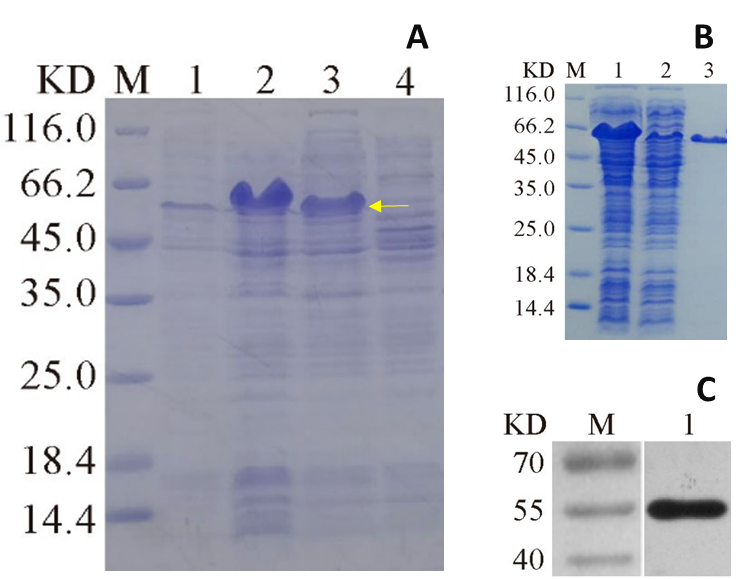


Figure S4. Expression and purification of *B. plantarii* TroK protein. (A) SDS-PAGE analysis of IPTG-induced expression of recombinant plasmid pCZN1-*troK* in *E. coli* Arctic Express DE3*.* 1. Non IPTG-inducible; 2. IPTG-induced; 3. Supernatant of cell debris; 4. Precipitates of cell debris; yellow arrow indicates TroK. (B) SDS-PAGE analysis of TroK purified by Ni-IDA-Sepharose Cl-6B column. 1. Pre-treatment samples; 2. Fraction by Ni-IDA Washing-Buffer; 3. Fraction by Ni-IDA Elution-Buffer. (C) Western Blot analysis of purified TroK. 1. Detection of TroK by using Mouse Anti-6X His tag (primary antibody, dilution ratio 1:1000) and Goat Anti-Mouse IgG H&L (HRP) (second antibody, dilution ratio 1:5000).


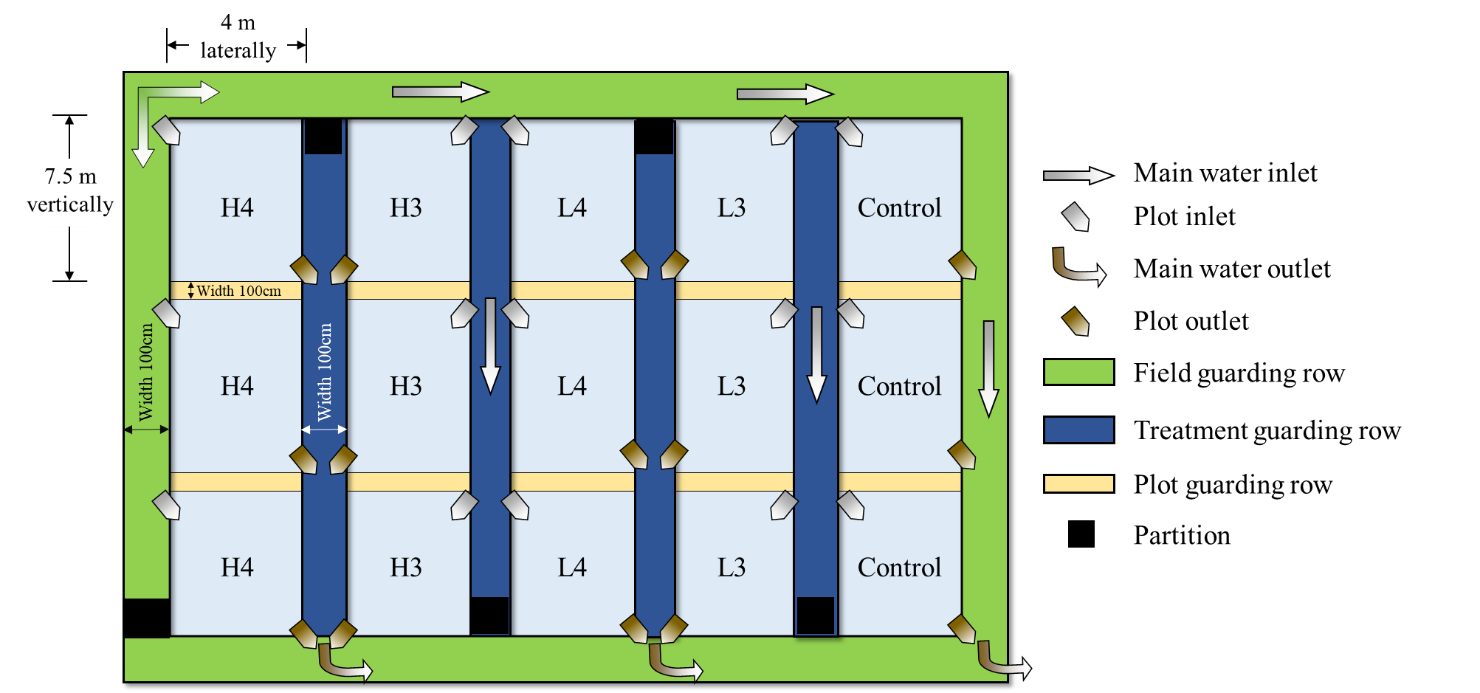


Figure S5. Schematic visualization of the field trial that was conduct to evaluate anti-virulence effects of thiazole. H and L indicate application dosages of zinc-thiazole at 1500 and 1875 mL/ha, respectively, while the numbers indicate application rates.


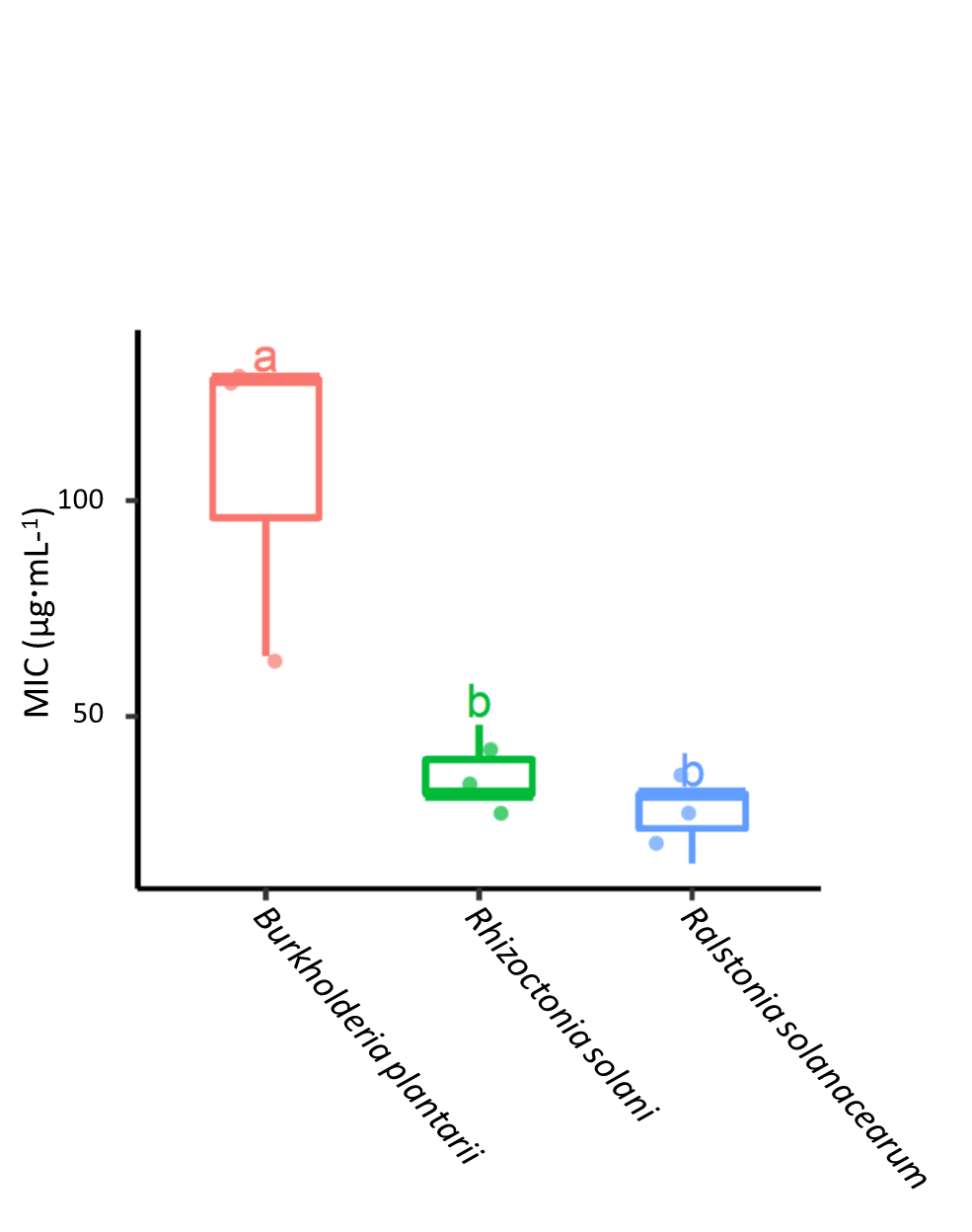


Figure S6. MIC of validamycin against three phytopathogens. Letters indicate significant difference by Student-Newman-Keuls test (*P* < 0.005).


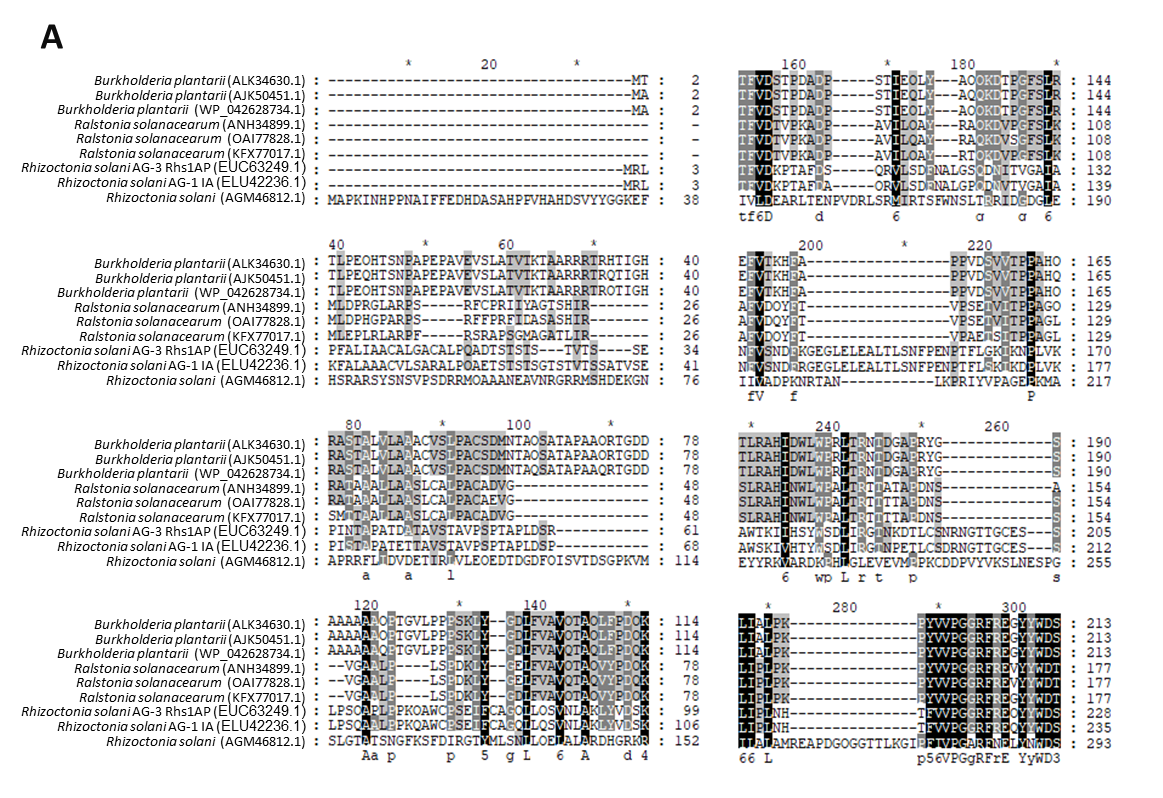


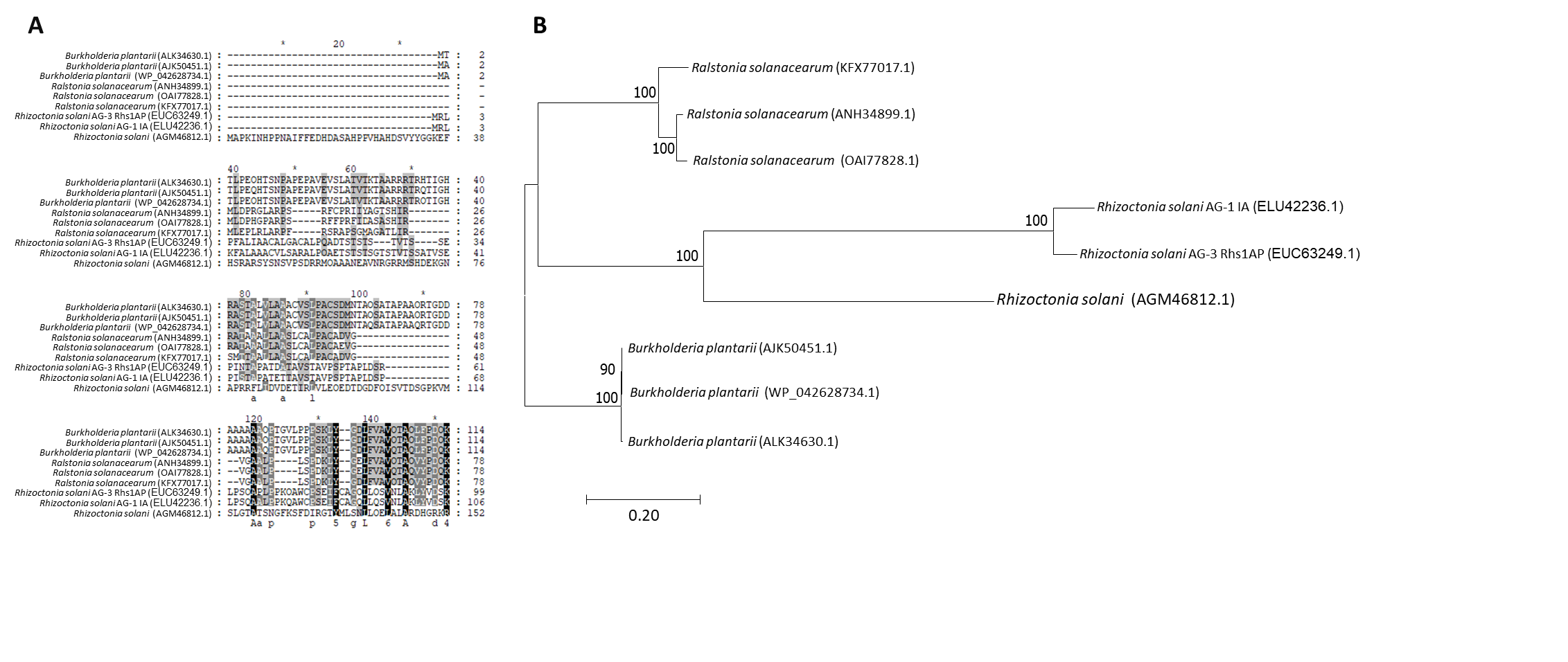


Figure S7. Comparison of trehalases in *Ralstonia solanacearum*, *Rhizoctonia solani* and *Burkholderia plantarii* in representative strains by alignment of conserved regions (A) and a corresponding phylogenetic analysis (B).

**
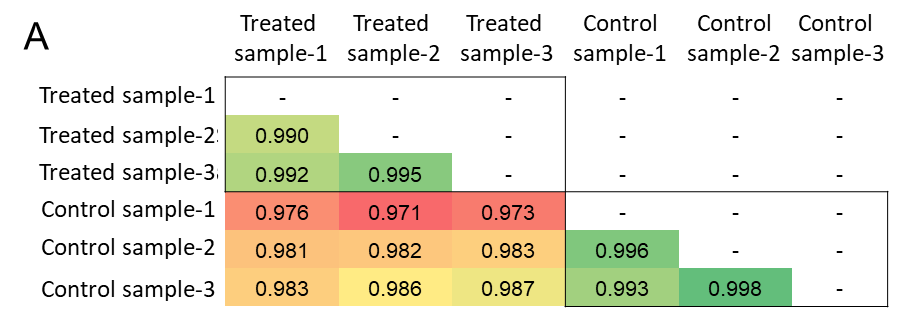
**

**
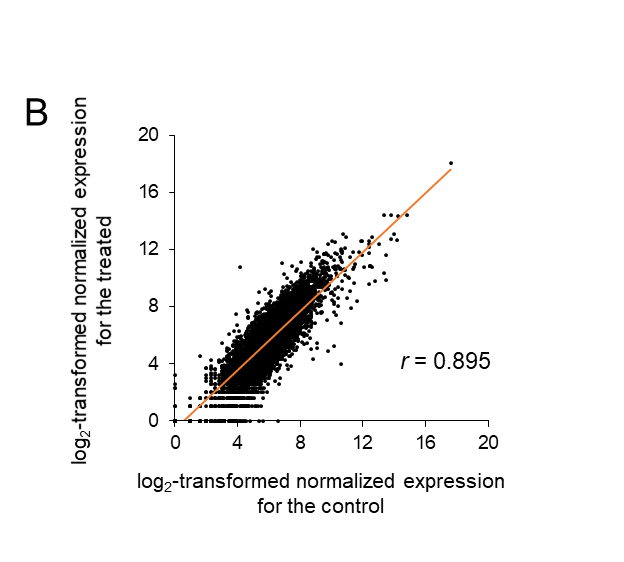
**

Figure S8. Repeatability assessment of RNA-seq experiments (A) and differential expression scatterplot of ATT-treated and control samples (B). The correlation coefficient *r* values are shown for each pair of RNA-seq experiments, with colors indicating the similarity degree between two samples. The orange line in the plot shows a linear regression of log_2_-transformed normalized gene expression levels of treated and control samples. ATT, 5-Amino-1,3,4-thiadiazole-2-thiol.


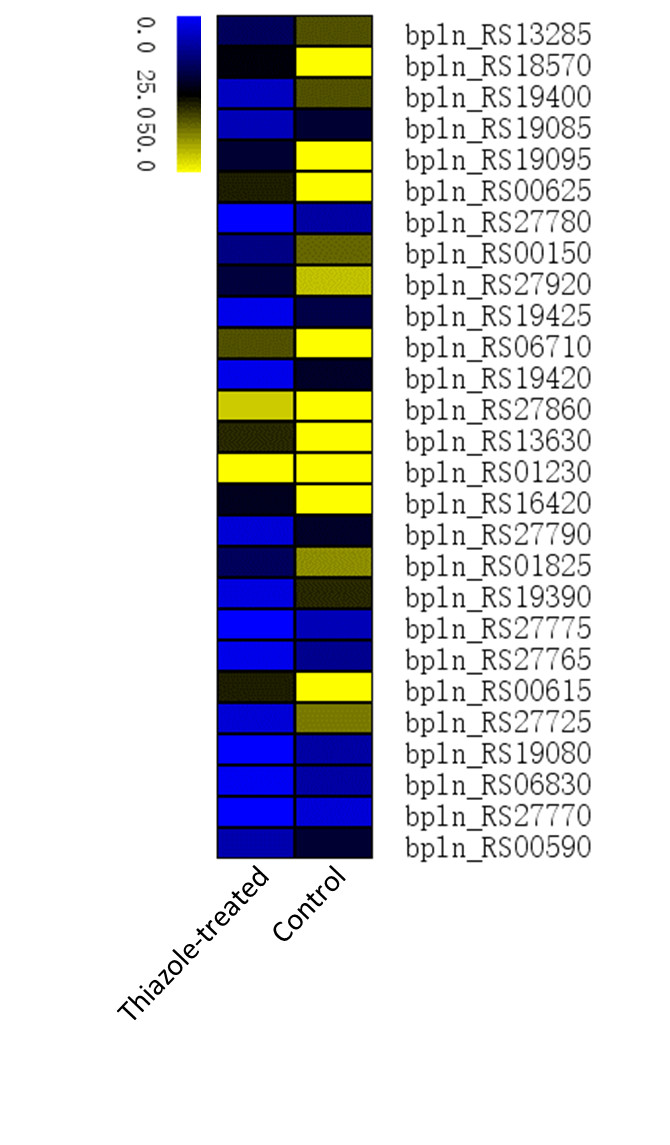


Figure S9. Differential analysis of BSS gene expression in *B. plantarii* exposed to ATT. Only BSS genes with significant changes according to Student’s t-test were included in the heatmap. BSS, bacterial secretion systems. ATT, 5-Amino-1,3,4-thiadiazole-2-thiol.

**
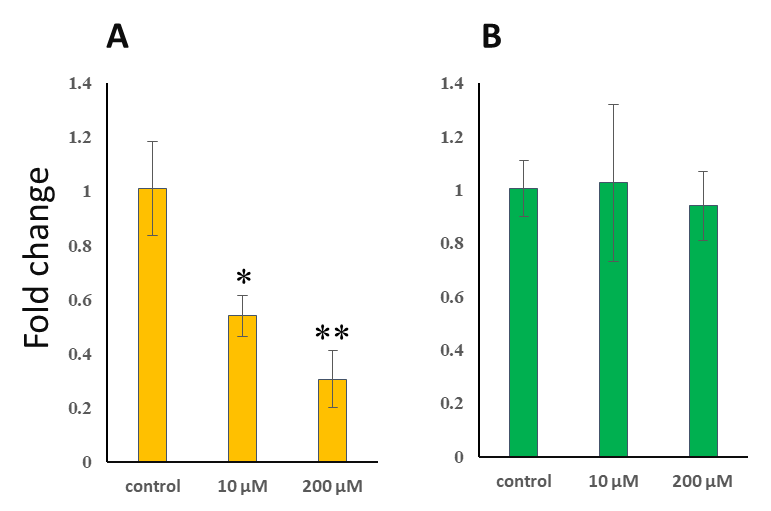
**

**
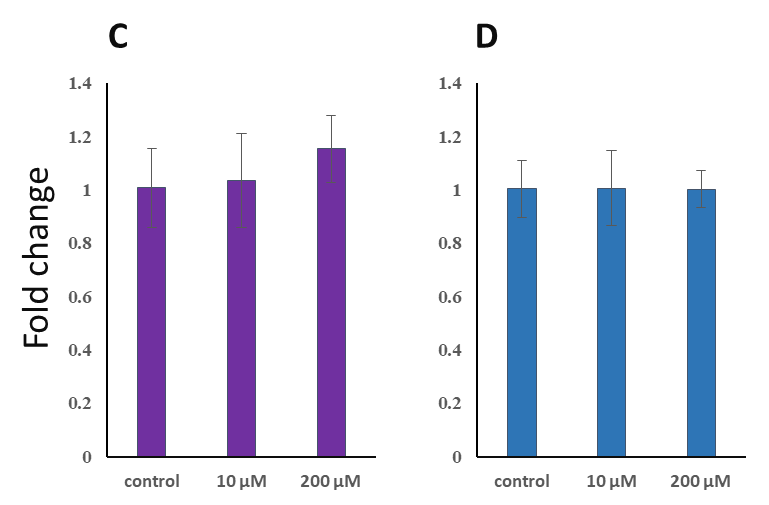
**

Figure S10. Gene expression in *B. plantarii* upon exposure to carot-4-en-9,10-diol. Expression of QS genes *plaI* (A) and TCS genes *troK* (B), *troR1* (C) and *troR2* (D) were measured in *B. plantarii* upon exposure to carot-4-en-9,10-diol at 20 and 100 μM. Values are means ± SD (shown as error bars) (n = 3).**P*＜0.01, ***P*＜0.001 by Student’s t-test. QS, quorum sensing. TCS, Two-component System.


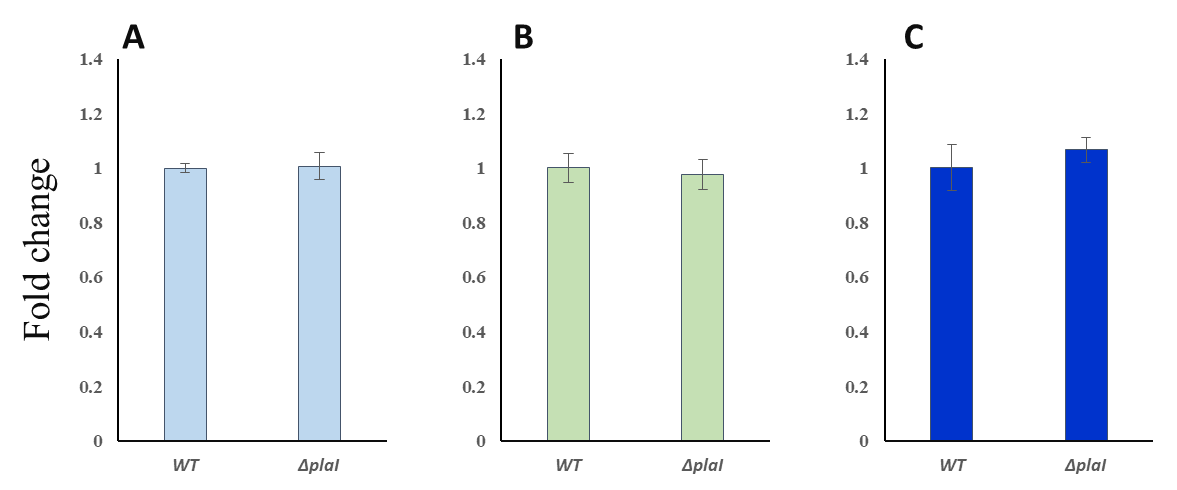


Figure S11. TCS gene expression in wild type (WT) and *plaI* mutant (*ΔplaI*) of *B. plantarii*. Expression of TCS genes *troK* (A), *troR1* (B) and *troR2*(C) were measured. Values are means ± SD (shown as error bars) (n = 3). TCS, Two-component System.


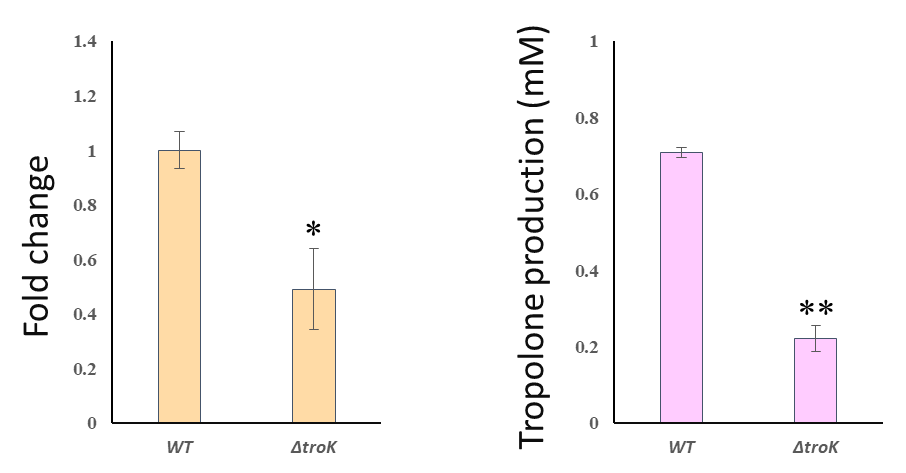
Figure S12. QS gene expression and tropolone secretion in wild type (WT) and *troK* mutant (*ΔtroK*) of *B. plantarii*. Values are means ± SD (shown as error bars) (n = 3). **P*＜0.01, ***P*＜0.001 by Student’s t-test. QS, quorum sensing.


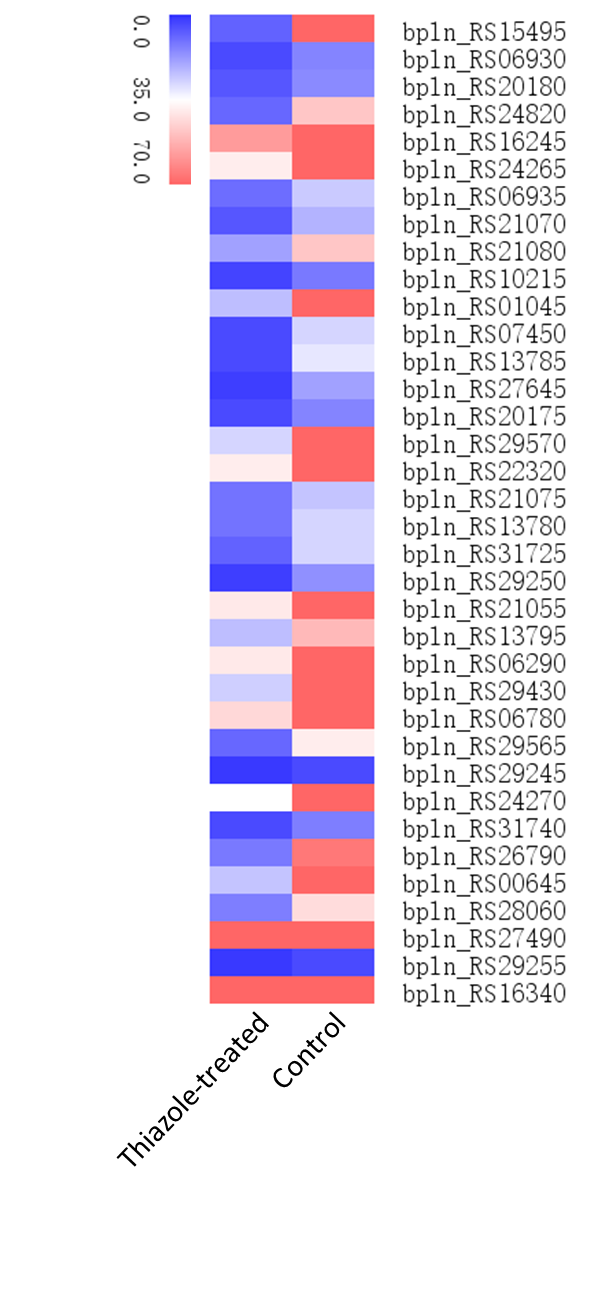


Figure S13. Differential analysis of TCS gene (non-KEGG enriched) expression in *B. plantarii* exposed to ATT. Only TCS genes with significant changes according to Student’s t-test were included in the heatmap. TCS, Two-component System. ATT, 5-Amino-1,3,4-thiadiazole-2-thiol.


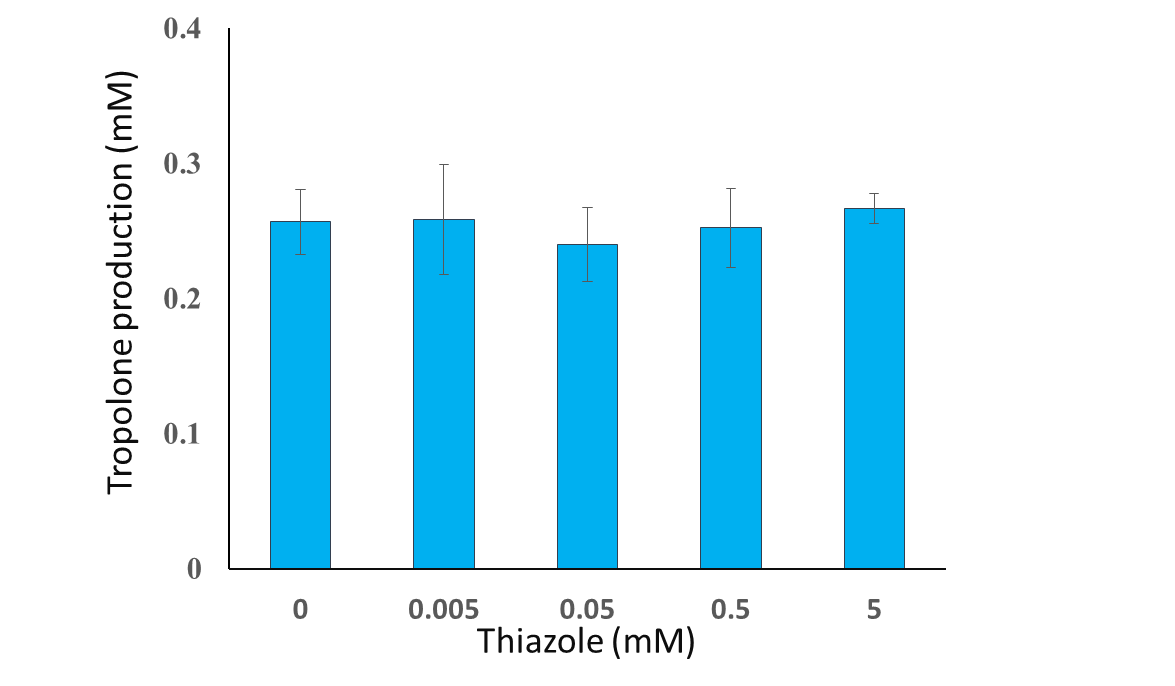


Figure S14. Tropolone secretion by *B. plantarii* (*ΔtroK*) exposed to a gradient of ATT. Values are means ± SD (shown as error bars) (n = 3). ATT, 5-Amino-1,3,4-thiadiazole-2-thiol.


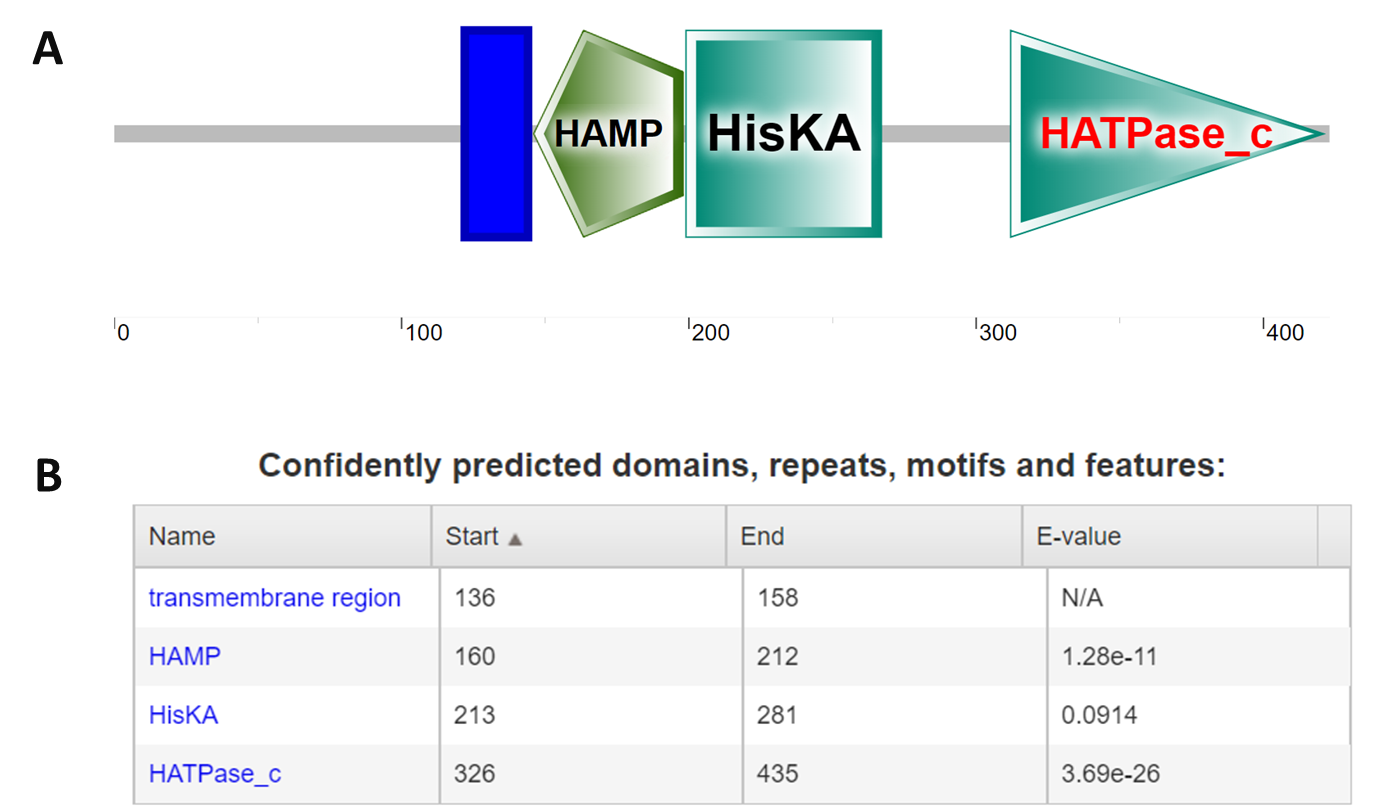


Figure S15. Analysis of the domain architecture of *B. plantarii* TroK. (A) Illustration of the domain structure of TroK. (B) Features of the predicted domains in TroK.


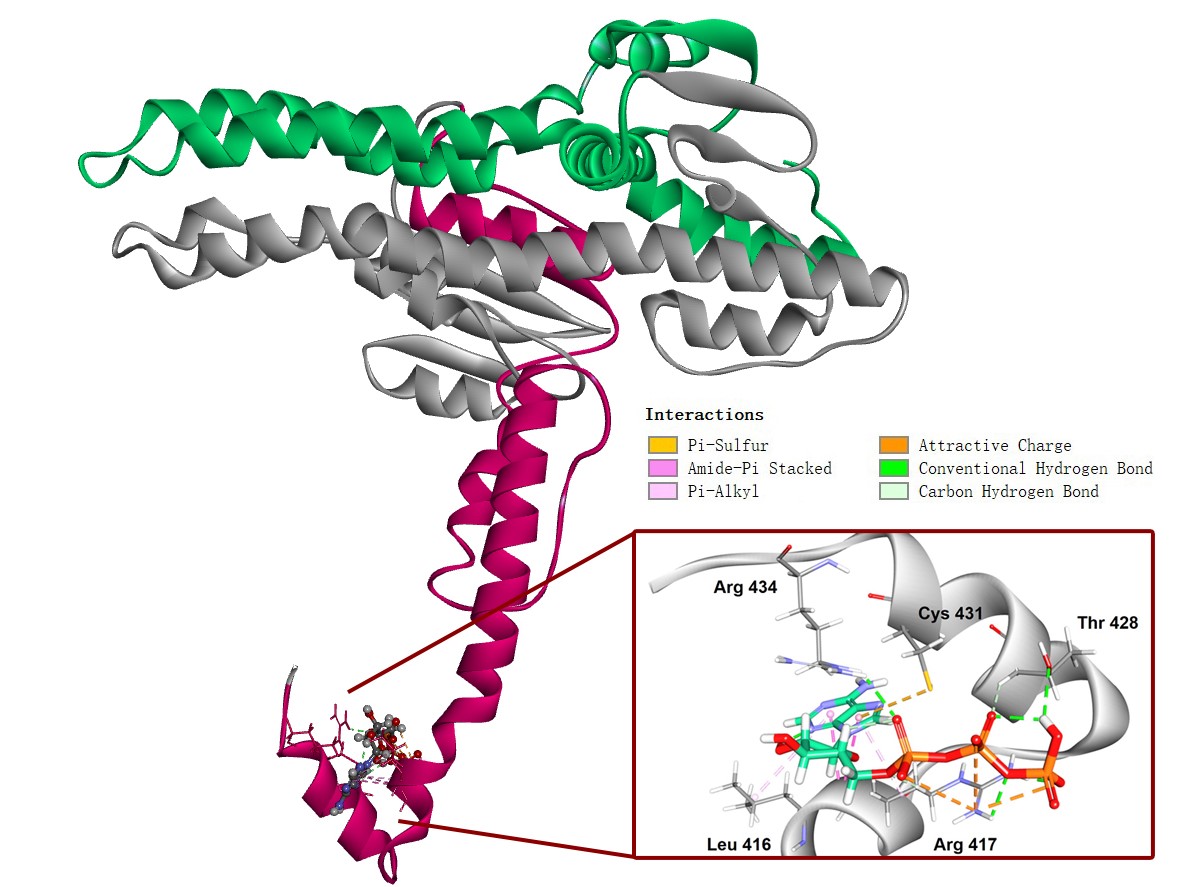


Figure S16. Molecular docking for binding sites involved in interaction between ATP and amino acid residues of TroK.


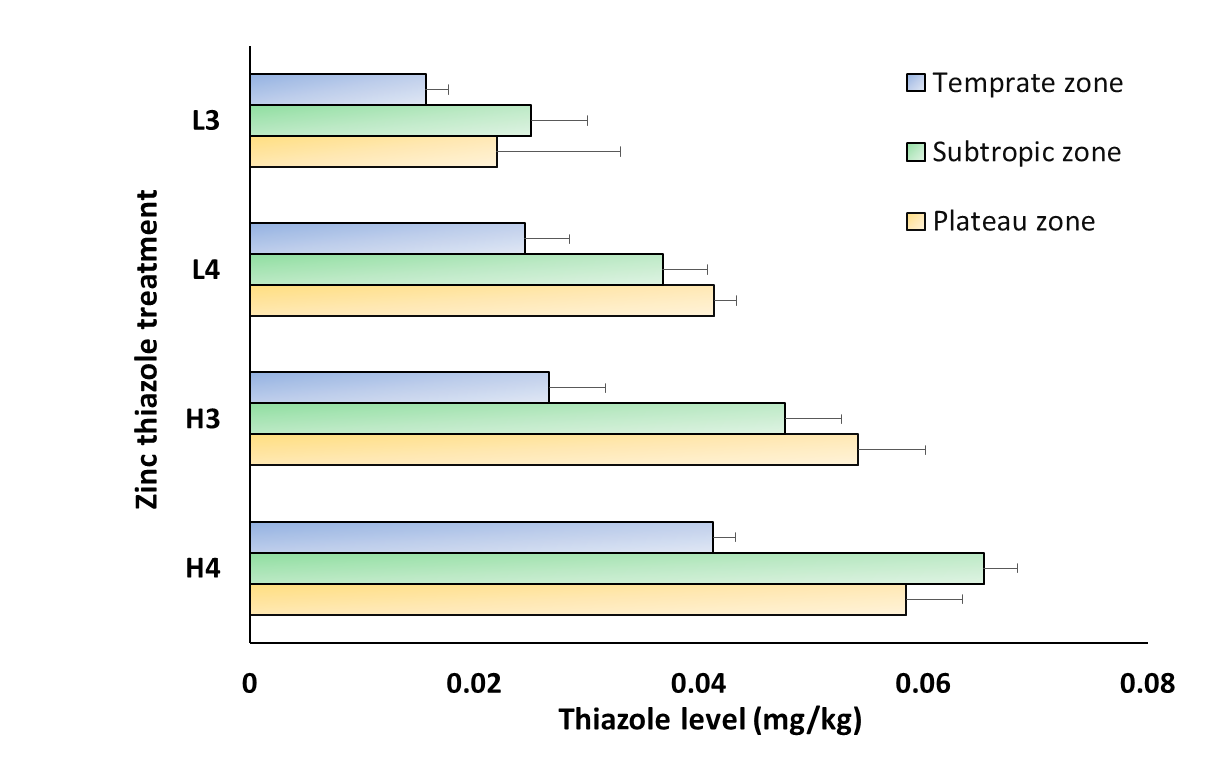


Figure S17. Quantification of ATT for dietary risk assessment. H and L indicates application dosages of zinc-thiazole at 1500 and 1875 mL/ha, respectively, and the following numbers indicate application rates. Values are means ± SD (shown as error bars, n = 10). ATT, 5-Amino-1,3,4-thiadiazole-2-thiol.


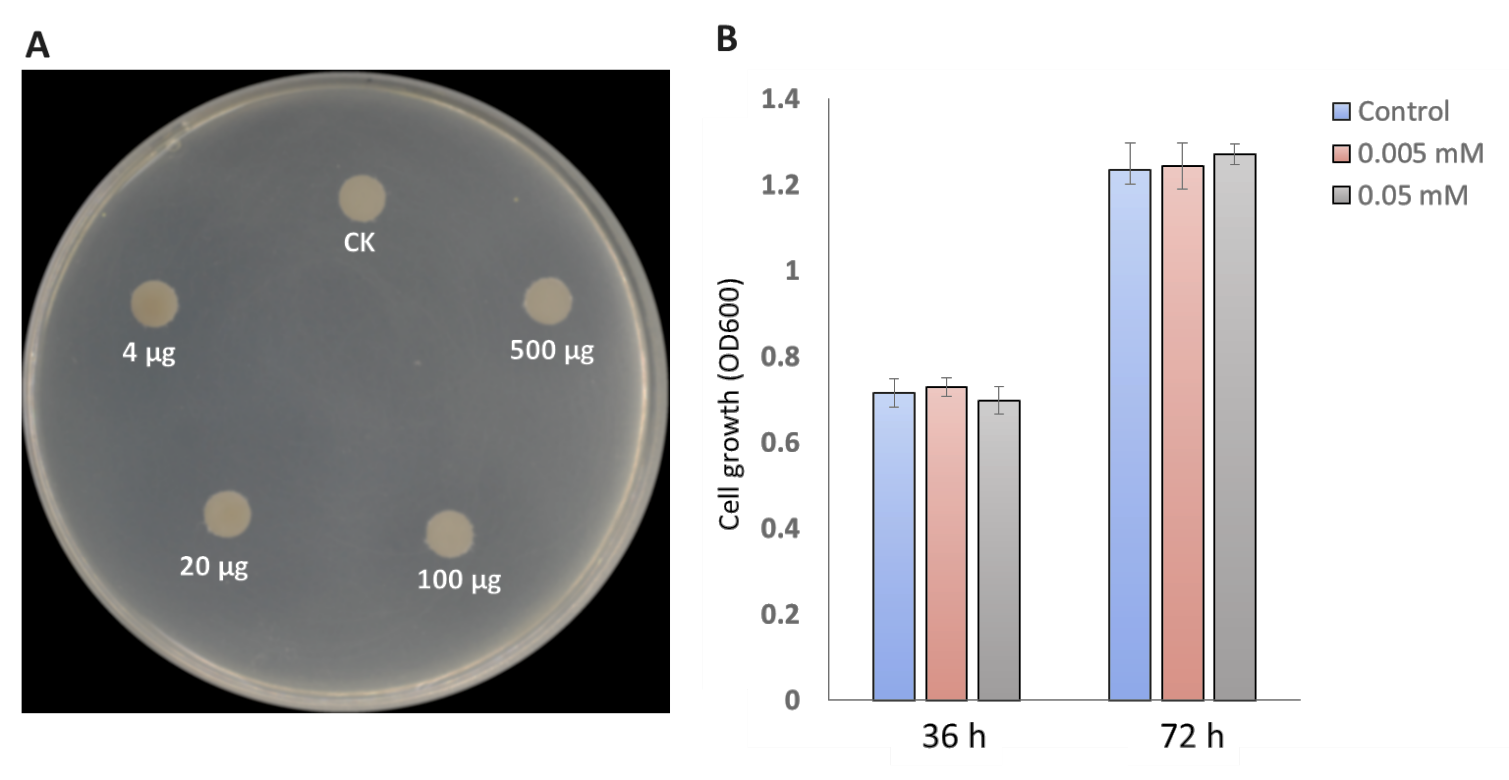


Figure S18. Impact of zinc-thiazole and ATT on *Sphingomonas melonis*. (A) The value neighboring the paper disc indicates the amount of zinc thiazole loaded (4-500 μg/disk). Control groups are indicated with ‘CK’, they were loaded with solvent only. No obvious halo area was observed around the zinc thiazole-charged paper discs in contrast to the control. (B) Cell growth of *S. melonis* exposed to gradient ATT. Values are means ± SD (shown as error bars) (n = 3). ATT, 5-Amino-1,3,4-thiadiazole-2-thiol.
